# Transcriptome profiling in rumen, reticulum, omasum, and abomasum tissues during the developmental transition of pre-ruminant to the ruminant in yaks

**DOI:** 10.3389/fvets.2023.1204706

**Published:** 2023-09-22

**Authors:** Yili Liu, Qi Min, Jiao Tang, Lu Yang, Xinxin Meng, Tao Peng, Mingfeng Jiang

**Affiliations:** ^1^Key Laboratory of Qinghai-Tibetan Plateau Animal Genetic Resource Reservation, College of Animal and Veterinary Sciences, Southwest Minzu University, Chengdu, China; ^2^Institute of Qinghai-Tibetan Plateau, Southwest Minzu University, Chengdu, China

**Keywords:** yak, stomach, DEmRNAs, STEM, WGCNA

## Abstract

The development of the four stomachs of yak is closely related to its health and performance, however the underlying molecular mechanisms are largely unknown. Here, we systematically analyzed mRNAs of four stomachs in five growth time points [0 day, 20 days, 60 days, 15 months and 3 years (adult)] of yaks. Overall, the expression patterns of DEmRNAs were unique at 0 d, similar at 20 d and 60 d, and similar at 15 m and adult in four stomachs. The expression pattern in abomasum was markedly different from that in rumen, reticulum and omasum. Short Time-series Expression Miner (STEM) analysis demonstrated that multi-model spectra are drastically enriched over time in four stomachs. All the identified mRNAs in rumen, reticulum, omasum and abomasum were classified into 6, 4, 7, and 5 cluster profiles, respectively. Modules 9, 38, and 41 were the most significant three colored modules. By weighted gene co-expression network analysis (WGCNA), a total of 5,486 genes were categorized into 10 modules. *CCKBR*, *KCNQ1*, *FER1L6*, and *A4GNT* were the hub genes of the turquoise module, and *PAK6*, *TRIM29*, *ADGRF4*, *TGM1*, and *TMEM79* were the hub genes of the blue module. Furthermore, functional KEGG enrichment analysis suggested that the turquoise module was involved in gastric acid secretion, sphingolipid metabolism, ether lipid metabolism, etc., and the blue module was enriched in pancreatic secretion, pantothenate and CoA biosynthesis, and starch and sucrose metabolism, etc. Our study aims to lay a molecular basis for the study of the physiological functions of rumen, reticulum, omasum and abomasum in yaks. It can further elucidate the important roles of these mRNAs in regulation of growth, development and metabolism in yaks, and to provide a theoretical basis for age-appropriate weaning and supplementary feeding in yaks.

## 1. Introduction

The yak (*Bos grunniens*) is a rare and valuable domesticated ruminant, which is mainly distributed in the Qinghai-Tibet Plateau and the adjacent high mountains above 3,000 meters. According to the National Catalogue of Livestock and Poultry Genetic Resources (2022 Edition), there are currently 21 yak species in China, including 19 local breeds and 2 cultivated breeds. They are mainly distributed in Sichuan, Tibet, Qinghai and other places in China, providing local herders with meat, milk, skin, hair, fuel and income. Compared with ordinary cattle, the meat, milk and processed products of yak have higher nutrient content and higher edible value, so yak is known as the “boat on the plateau” (1, 2). Maiwa yaks, one of the 19 local breeds, mainly rely on natural pasture with limited supplementation of concentrated feed. In nature, yaks graze on natural grassland all year round, but the lack of foraging resources poses a serious challenge to their survival in cold season (3). Therefore, yak ruminant stomach should have evolved a potential regulatory mechanism to adapt to the harsh plateau environment. Thus, transcriptome studies on yak ruminant stomach are of great significance to explore the development and metabolism-related genes and their expression patterns.

The ruminant stomach is composed of four compartments: rumen, reticulum, omasum, and abomasum, and each compartment has different physiological functions (4). It is well known that rumen epithelium contributes greatly to nutrient transport, absorption and metabolism of ruminant livestock (5, 6). Transcriptome sequencing is widely used to study the molecular mechanism of rumen development and metabolism (7–9). The gene expression pattern in the rumen is influenced by many factors, such as physiological state (10), RFI (residual feed intake, a measure of feed efficiency) (11), forage quality and particle size (8, 12), and age (13), etc. Ruminal epithelial cells are prone to acidosis, which further leads to rumen epithelial insufficiency, erosion and ulceration. The signal pathways in the rumen tissues of acidosis are enriched to cell signal transduction and morphogenesis, suggesting that rumen acidosis may affect the development of rumen epithelium in Holstein bull calves (10). Transcriptome analysis of rumen with different RFI has shown that the differentially expressed genes and pathways screened are mainly related to nutrient transport, cell growth and proliferation, immune function, inflammation, and apoptosis in beef cattle (11). In addition, nutrients and particle size are also important factors that alter the expression of genes participated in cell proliferation and apoptosis and complement complex. However, the related research on the reticulum, omasum and abomasum of yaks mainly focuses on the diversity of microorganisms, differences in flora and the crucial roles of these microorganisms in the whole growth and development (14, 15). Few reports indicate that the ruminant stomach, particularly the rumen and abomasum, show markedly different growth and developmental processes after birth and subsequent ruminant stages in Holstein cattle (16). From birth to adulthood, the four stomachs of yaks have significant changes in size and function, which play important physiological functions, respectively (4). As far as we know, there are almost no reports on the combined analysis of gene expression patterns in rumen, reticulum, omasum, and abomasum tissues of yaks at different developmental stages.

In this study, to further elucidate the molecular basis of physiological functions of rumen, reticulum, omasum and abomasum, we first compared mRNA expression levels of ruminant stomach at five developmental stages. At the transcriptome level, we systematically analyzed the differentially expressed genes, spatiotemporal expression patterns, and potential biological functions in four stomach tissues of yaks. STEM analysis was used to investigate the dynamic expression patterns of mRNAs, and a network diagram of rumen-reticulum-omasum-abomasum interactions was constructed with significant changes over time. In addition, screening of mRNAs affecting the development and metabolism of the four stomachs during the whole developmental stage of yaks can significantly help to clarify biological and metabolic functions and relative importance. This finding will help researchers to further clarify the physiological function and regulatory mechanism of ruminant stomach in yaks, and lay a theoretical foundation for subsequent studies on the efficient and precise regulation of ruminant stomach in terms of development and metabolism, and contribute to the further development of animal husbandry on the Tibetan Plateau.

## 2. Materials and methods

### 2.1. Animals and sample collection

All experimental procedures on live animals have been strictly reviewed and approved by the Institutional Animal Care and Use Committee at Southwest Minzu University (Chengdu, Sichuan, China), and all the experiments were carried out in accordance with the requirements of the directory of the Ethical Treatment of Experimental Animals of China. All Maiwa yaks were raised in Hongyuan County, Aba Tibetan and Qiang Autonomous Prefecture, Sichuan Province. All yaks were naturally grazed without feed supplementation, and calves fed on breast milk and natural forage. Fifteen Maiwa yaks were randomly selected from five different growth stages, including 0 d (0 day of age), 20 d (20 days of age), 60 d (60 days of age), 15 m (15 months of age), and adult (3 years of age). Each age group consisted of 3 healthy yaks with similar body weight, and the gender was selected randomly. Because of the large size of yaks, it is not suitable to use anesthetic in the sampling process, so a more extensive and humane method of electric shock was adopted in this experiment. To ameliorate suffering, yaks were electrically stunned (120 V dc, 12 s) before exsanguination, and sacrificed by bloodletting through the carotid artery and jugular vein while in a coma. After slaughter, the contents of each stomach chamber were emptied immediately and washed with saline several times until the attached contents are completely removed. Tissue blocks of approximately 1 cm × 1 cm × 1 cm were immediately taken from 4 stomachs with high-temperature sterilizing scissors, washed with normal saline and DEPC water successively, then put into the RNA-free frozen storage tube and frozen in liquid nitrogen until RNA was extracted.

### 2.2. Organism index measurement

All Maiwa yaks were accurately weighed and recorded before slaughter. After slaughter, the ruminant stomach was removed, and the connected parts of rumen, omasum, reticulum and abomasum were ligated, and the four gastric compartments were separated. In the rumen, we found no solid contents in the 0 d group, very little pasture and a lot of milk in the 20 d group, some pasture and a lot of milk in the 60 d group, and only pasture in the 15 m and adult groups. The weights of the rumen, reticulum, omasum and abomasum were separately measured and recorded after completely emptying digesta and fluid. The stomach index was calculated using the following formula: Stomach index = single stomach weight (g)/ruminant stomach weight (g) × 100%.

### 2.3. RNA extraction

Total RNA was extracted from each sample using the mirVana miRNA Separation kit (Ambion-1561) according to the manufacturer’s instructions. RNA concentration and integrity were measured and assessed using the NanoDrop 2000 (Thermo Fisher Scientific) and the Agilent 2100 Bioanalyzer (Agilent Technologies, Santa Clara, CA, United States). The samples with RNA Integrity Number (RIN) ≥ 7 were further analyzed.

### 2.4. Library construction and sequencing

The libraries were constructed following the manufacturer’s protocol at Shang-hai OE Biotech (Shanghai, China), using TruSeq Stranded mRNA LTSample Prep Kit (Illumina, San Diego, CA, United States). In short, these mRNA samples were enriched with the Oligo (dT) magnetic beads and fragmented using the breaking reagent. Then first-strand cDNA and second-strand cDNA were successively synthesized and purified. The purified double-stranded cDNA was end-repaired, A-tailed, ligated to sequencing adapters, amplified and purified. These libraries were purified using AMPure XP beads and analyzed for purity and size by Agilent 2100 Bioanalyzer. These libraries were sequenced on the Illumina HiSeq X Ten and 150 bp paired-end reads were obtained.

### 2.5. Read mapping and transcriptome assembly

Trimmomatic software was used to process the raw data (raw reads) for quality control (17). The clean reads were obtained by removing the raw reads containing ploy-N and the low-quality reads. The high-quality clean reads were then mapped to the yak reference genome (GCF_000298355.1) using hisat2 (18) (Version 2.2.1).^1^ The cufflink software was used to calculate the FPKM value of each gene (19, 20), and the htseq-count software was used to count the number of reads mapped to each gene (21). For quantification of transcription levels, bowtie2 (22) and eXpress (23) were used to calculate FPKM and read counts value of each transcript. The StringTie software was adopted to reassemble the reads (24). On this basis, using cuffCompare software, the reference genome was compared with the existing annotated genes to identify the gene structure extension and novel transcripts. The ASprofile was used to perform the alternatively splicing analysis of differentially regulated transcripts isoforms or exons (25). SNP and INDEL were called using samtools (26) and bcftools (27), and the details were shown on samtools webpage.^2^ Then the effects of variants on genes were annotated and predicted by the sniff (28).

### 2.6. Differential expression analysis and functional enrichment

Based on the DESeq (2012) (29) R package functions estimateSizeFactors and nbinomTest, differential expression analysis was performed and differentially expressed genes (DEGs) were identified. Adjusted *p* value < 0.05 and fold Change ≥ 2 or fold Change ≤ 0.5 was set as the threshold for significantly differential expression. To explore gene and transcripts expression patterns, hierarchical cluster analysis of DEGs was executed. GO enrichment and KEGG pathway enrichment of DEGs were analyzed according to the methods in the references (30).

### 2.7. Time-series analysis

The STEM clustering algorithm was used to explore the relationship between temporal gene expression patterns and yak stomach tissues (rumen, reticulum, omasum, and abomasum) during the five developmental stages (31). Time-series analysis mainly studied the dynamic expression of genes, measuring a range of processes strongly related to developmental stages. Significantly enriched model profiles were displayed in different colors (Benjamini-Hochberg-adjusted *p*-values ≤ 0.05). The corrected *p*-values were sorted from small to large. Colored model profiles (except white) indicated that the temporal trends of mRNAs were statistically significant. Model profiles with the same color belonged to the same cluster.

### 2.8. Co-expression networks

The co-expression networks of rumen-reticulum-omasum-abomasum mRNAs were established by the WGCNA package of R software through the developmental stages (32). After eliminating samples with outliers samples, the Pearson’s correlation coefficient between any two genes was determined in the gene set, and the correlation coefficients matrix was builded. The appropriate threshold value (β value) was selected to measure the weighted power exponent of the correlation coefficient matrix, and the adjacency matrix was established. Next, the topological overlap matrix was established and employed to the connections between different genes. Based on related traits, the gene modules were preliminarily divided by hierarchical clustering analysis, and the eigengenes were obtained. According to the similarity of eigengenes, these modules were combined to form a final module for further study (33). The module membership (MM) was calculated by the WGCNA function signed KME, which correlated the module eigengene (ME) with gene expression values, thereby quantifying how close a gene was to a given module. Gene significance (GS) referred to the correlation between a single gene and a biological trait. The summation of adjacency performed for all genes in a particular network was calculated as the intramodular connectivity (K.in). We first selected genes with GS > 0.25 and MM > 0.95. Then, we screened for genes with the top50 of the number of gene interaction nodes as hub genes. Visualization analysis of gene interaction network was performed using cytoscape software v3.8.2.

### 2.9. Quantitative real-time PCR analysis

To verify the reliability of RNA-seq data, 16 genes were randomly selected for RT-qPCR quantification. Total RNA was isolated from the rumen, reticulum, omasum and abomasum of yaks according to the instructions of the mirVana miRNA Isolation Kit (Ambion). Reverse transcription was used to synthesize cDNA according to the instructions of the PrimeScript RT reagent Kit with gDNA Eraser (TaKaRa, Dalian, China). The transcription levels of mRNAs (*S100A12*, *KRT4*, *KRT6A*, *ACTG2*, *A2ML1*, *S100A9*, *FTH1*, *DES*, *RPLP0*, *ANXA1*, *RPS3A*, *TFF1*, *GKN1*, *ATP4B*, *PGC*, and *RPS8*) were verified by LightCycler 96 System (Roche, United States). The forward and reverse primers for RT-qPCR are shown in Supplementary Table S1. RT-qPCR was performed in a 96-well plate containing 20 μL mixture per well, including 10 μL of TB Green Premix Ex Taq^™^ II (Tli RNaseH Plus) (TaKaRa, Dalian, China), 2.0 μL of cDNA template, 0.5 μL of forward and reverse primer (10 μM), and 7.0 μL of RNase Free ddH2O. The running conditions of RT-qPCR were set as follows: 95°C for 30 s (pre-denaturation), 40 cycles of amplification (95°C for 5 s, 60°C for 30 s) and 95°C for 5 s, 60°C for 1 min (Melting Curves). For each time point, the gene validation was conducted in triplicate. The 2^−∆∆Ct^ method was used to calculate the expression level of each verified gene at each time point.

## 3. Results

### 3.1. Change in yak stomach index

**Figure 1:**
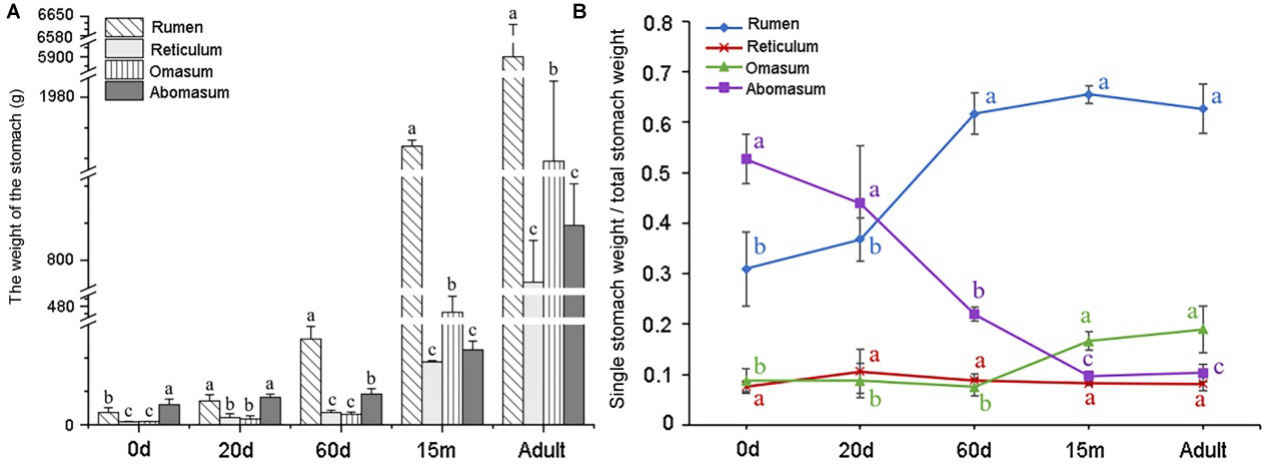
Analysis of weight changes in four stomachs of yaks at five developmental stages. **(A)** Weight variation of four stomachs in yak at different ages. *N* = 3/group, the abscissa shows the age, and the ordinate shows the weight (g). **(B)** Analysis of the ratio of individual stomach weight to total stomach weight of yaks in different stages. *N* = 3/group, the abscissa represents the age, and the ordinate represents the ratio.

The increase in volume and weight of the ruminant stomach is one of the important indexes to measure the development stage of yak. This study measured the weight of four stomachs at different developmental stages. At 0 d, the weight of the abomasum was the largest, followed by the rumen, and the weight of the abomasum was markedly heavier than that of the other three stomachs (*p* < 0.05, Figure 1A). At 20 d, the weight of abomasum was almost equal to that of rumen, and there was no significant difference between them (*p* > 0.05), but the weight of abomasum was significantly heavier than that of reticulum and omasum (*p* < 0.05, Figure 1A). At 60 d, the weight of rumen was the heaviest, which was significantly heavier than that of abomasum (*p* < 0.05), and the abomasum weight was significantly heavier than that of reticulum and omasum (*p* < 0.05, Figure 1A). At 15 m, the weight of rumen was significantly heavier than that of omasum (*p* < 0.05), and the weight of reticulum and abomasum was the lightest. At adult group, the weight of rumen was the heaviest, which was significantly heavier than that of omasum (*p* < 0.05), and the weight of abomasum was heavier than that of reticulum (*p* > 0.05, Figure 1A). According to the yak stomach index at 0 d and 20 d, the ratio of abomasum was higher than that of the forestomach. At 60 d, the ratio of the rumen was the highest, followed by the abomasum. At 15 m and adult groups, the ratio of rumen was the largest, followed by omasum, abomasum and reticulum (Figure 1B).

### 3.2. Sequencing and *de novo* transcriptome assembly

To identify the dynamic changes of four stomachs in transcriptome profiles at different growth stages, the transcriptome sequencing of 60 samples was performed, and 2906.51 Mb clean reads was gained (Supplementary Table S2). For each sample, the effective data volume was distributed in 6.08–7.34 G, the Q30 base distribution was 93.36–96.03%, and the average GC content was 51.05%. The genome alignment of each sample was gained by aligning the reads to the reference genome, and the alignment rate was 89.63–96.59%. The above results indicated that the sequencing data were qualified and in-depth enough for further analysis.

### 3.3. Expression patterns of DEmRNAs in rumen tissue

**Figure 2:**
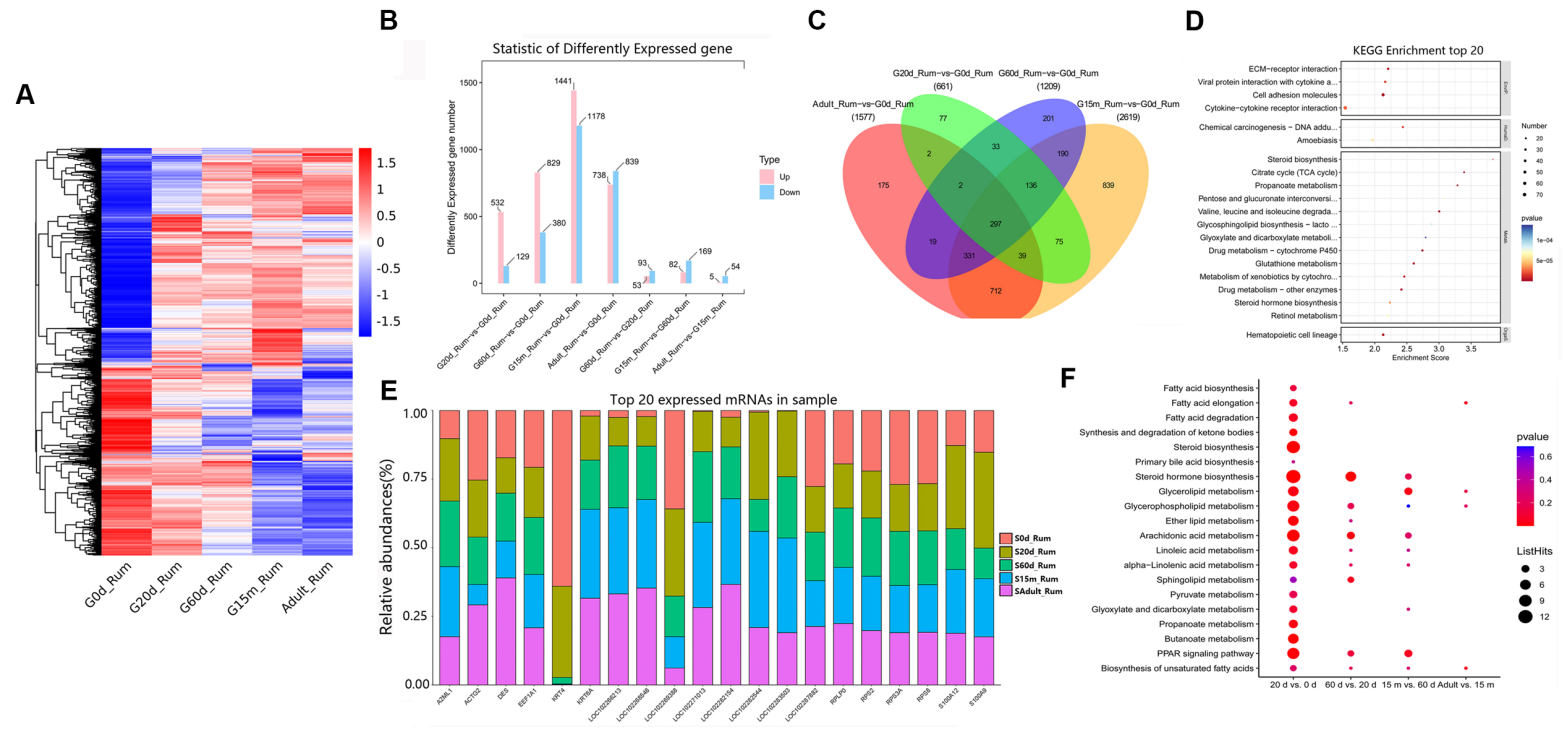
Expression patterns of DEmRNAs in rumen. **(A)** Heatmap of DEmRNAs in the seven compared groups. **(B)** Number of DEmRNAs in rumen. **(C)** The number of common DEmRNAs in rumen. **(D)** KEGG pathway analysis of common DEmRNAs in rumen. The top 20 enriched KEGG pathways ranked by *p-*values are shown. **(E)** Top 20 mRNAs expressed in rumen. **(F)** KEGG enrichment bubble plot of DEmRNAs involved in fatty acid metabolism in four consecutive groups.

To understand the regulatory roles of mRNAs of rumen, reticulum, omasum and abomasum, we analyzed the expression profile of mRNAs in four stomachs at 0 d, 20 d, 60 d, 15 m and adult. The study was able to evaluate the dynamic changes of mRNA expression from non-ruminant to ruminant stage, and screen and identify important mRNAs related to the growth and metabolism in ruminant stomach of yaks. First, we designed two comparative methods, namely, closed groups (20 d vs. 0 d, 60 d vs. 0 d, 15 m vs. 0 d and adult vs. 0 d), and consecutive groups (20 d vs. 0 d, 60 d vs. 20 d, 15 m vs. 60 d, and adult vs. 15 m) to characterize the DEmRNAs during development. Among these comparison groups in the rumen, 3,240 mRNAs were defined as DEmRNAs (Figure 2A).

We attempted to evaluate the potential functions of DEmRNAs between two closed time points in the rumen. Compared with 0 d, 532, 829, 1,441, and 738 mRNAs were up-regulated at 20 d, 60 d, 15 m and adult groups, respectively (Figure 2B). KEGG pathway analysis showed that, compared with 0 d, the significantly up-regulated pathways in the other four stages are all concerned with lipid metabolism, carbohydrate metabolism, and immune response including such processes as steroid biosynthesis, butanoate metabolism, propanoate metabolism, hematopoietic cell lineage (Supplementary Table S3). Among these DEmRNAs, 297 were identified as common DEmRNAs throughout rumen development (Figure 2C, Supplementary Table S4). These common DEmRNAs played essential roles in rumen development, immune response, carbohydrate metabolism and lipid metabolism. KEGG pathway analysis indicated that these common DEmRNAs were primarily enriched in ECM-receptor interaction, viral protein interaction with cytokine and cytokine receptor, cell adhesion molecules, propanoate metabolism, steroid biosynthesis, and hematopoietic cell lineage, etc. (Figure 2D, Supplementary Table S5).

To study the role of DEmRNAs in transforming rumen function, in addition to 20 d vs. 0 d, three comparison groups including 60 d vs. 20 d, 15 m vs. 60 d and adult vs. 15 m were added. Compared to 0 d, the up-regulated pathways at 20 d were primarily related to steroid biosynthesis, steroid hormone biosynthesis, pentose and glucuronate interconversions, hematopoietic cell lineage, butanoate metabolism, mineral absorption, cell cycle, and the down-regulated pathways were observed to be principally related to MAPK signaling pathway, cortisol synthesis and secretion, central carbon metabolism in cancer, and human T-cell leukemia virus 1 infection. Compared to 20 d, up-regulated pathways at 60 d were chiefly related to steroid hormone biosynthesis, drug metabolism—cytochrome P450, retinol metabolism, and pentose and glucuronate interconversions, and the down-regulated pathways were primarily related to aldosterone-regulated sodium reabsorption, pancreatic secretion and sphingolipid metabolism. Compared to 60 d, the up-regulated pathways at 15 m were mainly involved in kaposi sarcoma-associated herpesvirus infection, IL-17 signaling pathway, glycine, serine and threonine metabolism, and NF-kappa B signaling pathway. The down-regulated pathways were primarily involved in such metabolic pathways as ECM-receptor interaction, and protein digestion and absorption. Compared to 15 m, the significantly up-regulated pathways at adult were primarily related to antifolate resistance, ABC transporters, bile secretion, purine metabolism and cAMP signaling pathway, and the down-regulated pathways were engaged mainly in such immune processes as pertussis, systemic lupus erythematosus, and complement and coagulation cascades (Supplementary Table S6). These up- and down-regulated mRNAs at different development stages may be involved in regulating the beginning or ending of growth and development, and/or important physiological processes at specific developmental stages. We also focused on the changing rules of the 20 most abundant mRNAs in the rumen. Notably, several of the most abundant mRNAs in the rumen originate from protein-coding genes with pivotal roles in rumen growth, immune response, and translation (e.g., *S100A12*, *S100A9*, *RPS2*, *KRT4*, *RPS8*, *KRT6A*, *DES*, *A2ML1*, *EEF1A1*, *ACTG2*, *RPS3A*, and *RPLP0*) (Figure 2E, Supplementary Table S7). KEGG analysis was performed to further explore the switching of fatty acid metabolism-related functions at different developmental stages in the rumen. The results showed that 20 days is an important metabolic time point, and many fatty acid pathways are significantly enriched at 20 days, and there are different degrees of enrichment at 60 days, 15 months and adult groups (Supplementary Figure S1, Figure 2F). There were no differences in some metabolic pathways in the 60 d vs. 20 d, 15 m vs. 60 d and adult vs. 15 m groups, including fatty acid biosynthesis, fatty acid elongation, synthesis and degradation of ketone bodies, steroid biosynthesis, pyruvate metabolism, propanoate metabolism, synthesis and degradation of ketone bodies, steroid biosynthesis, pyruvate metabolism, propanoate metabolism, butanoate metabolism (Figure 2F).

### 3.4. Expression patterns of DEmRNAs in reticulum tissue

**Figure 3:**
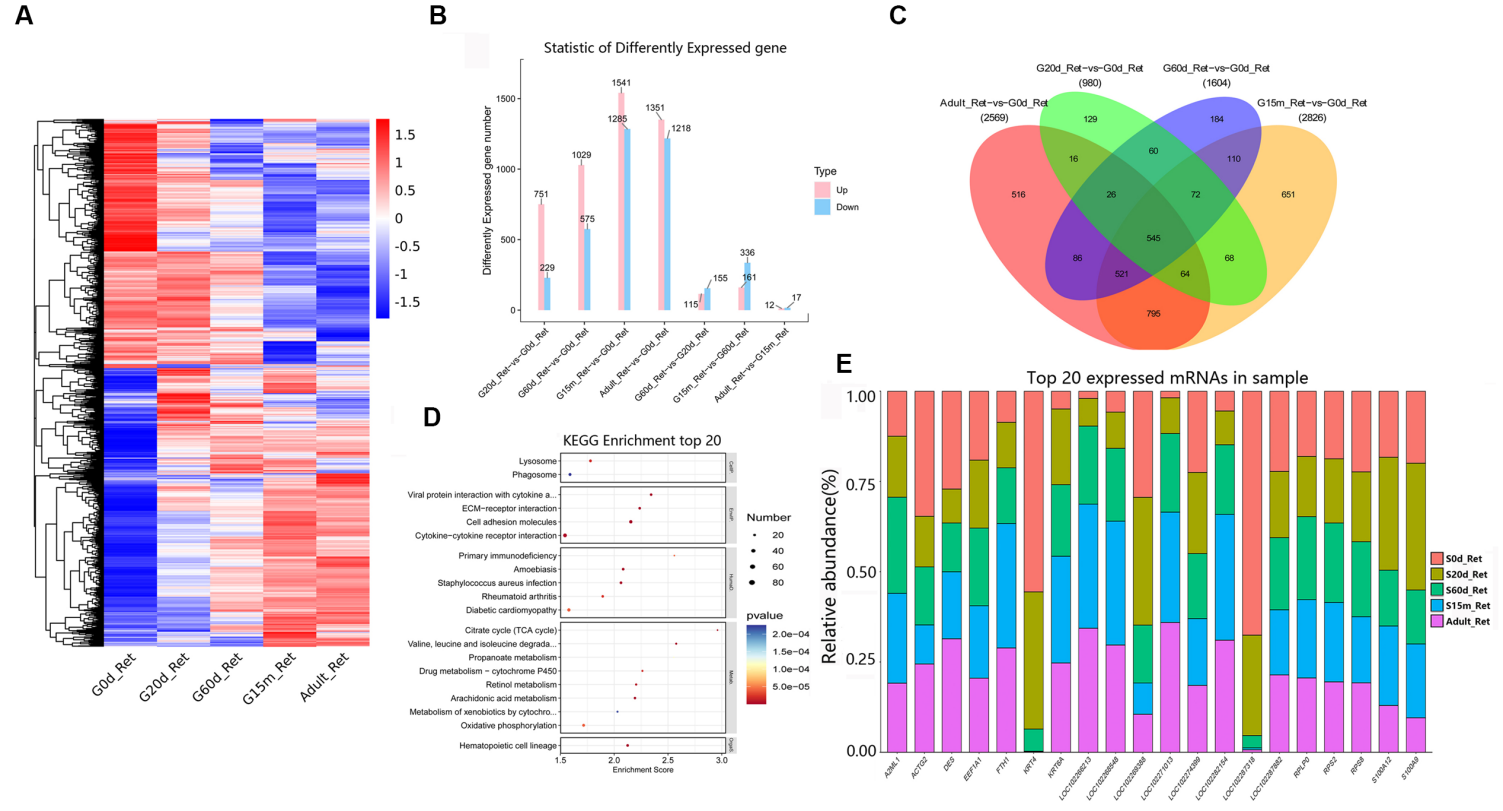
Expression patterns of DEmRNAs in reticulum. **(A)** Heatmap of DEmRNAs in the seven compared groups (20 d vs. 0 d; 60 d vs. 0 d; 15 m vs. 0 d; adult vs. 0 d; 60 d vs. 20 d; 15 m vs. 60 d; and adult vs. 15 m groups). **(B)** The number of DEmRNAs. **(C)** The number of common DEmRNAs. **(D)** KEGG pathway analysis of common DEmRNAs. The top 20 enriched KEGG pathways ranked by *p*-values are shown. **(E)** The top 20 expressed mRNAs.

The reticulum acts mechanically to reduce the ingesta to fine particles further. In the seven comparison groups, we identified 3,968 DEmRNAs to gain an overview of DEmRNAs in the reticulum (Figure 3A). Among these closed time points, compared to 0 d, we detected 751, 1,029, 1,541, and 1,351 DEmRNAs upregulated at 20 d, 60 d, 15 m and adult (Figure 3B). At these four closed time points, KEGG analysis showed that the up-regulated pathways were substantially enriched in immune-related signaling pathways, including hematopoietic cell lineage, cell adhesion molecules, cytokine-cytokine receptor interaction, viral protein interaction with cytokine and cytokine receptor, etc. Additionally, between 20 d and 60 d, significantly up-regulated pathways were involved in retinol metabolism, bile secretion, pentose and glucuronate interconversions, ascorbate and aldarate metabolism, steroid hormone biosynthesis, and butanoate metabolism, and the down-regulated pathways were primarily involved in IL-17 signaling pathway, viral protein interaction with cytokine and cytokine receptor, neomycin, kanamycin and gentamicin biosynthesis, african trypanosomiasis, and glycerophospholipid metabolism. Compared to 60 d, up-regulated pathways at 15 m were predominantly related to metabolic pathways including mineral absorption, steroid hormone biosynthesis, and arachidonic acid metabolism, and the down-regulated pathways are mainly related to ECM-receptor interaction, protein digestion and absorption, PI3K-Akt signaling pathway, etc. Compared to 15 m, the up-regulated pathways at adult were primarily related to hematopoietic cell lineage, Ras signaling pathway, MAPK signaling pathway, and PI3K-Akt signaling pathway, and the down-regulated pathways were primarily related to peroxisome, primary bile acid biosynthesis, steroid biosynthesis, arginine and proline metabolism, and arachidonic acid metabolism (Supplementary Table S8).

During the entire development process, compared to 0 d, 545 were regarded as the common DEmRNAs of the reticulum (Figure 3C), showing that the KEGG pathways were chiefly focused on lysosome, phagosome, viral protein interaction with cytokine and cytokine receptor, ECM-receptor interaction, cell adhesion molecules, cytokine-cytokine receptor interaction, primary immunodeficiency, TCA cycle, valine, leucine and isoleucine degradation, propanoate metabolism, and hematopoietic cell lineage, etc. (Figure 3D, Supplementary Table S9). Furthermore, among the 20 most abundant mRNAs expressed in the reticulum, several mRNAs mainly derived from protein-coding genes with the critical roles in reticulum development, immune response and mineral absorption are highly expressed (e.g., *S100A12*, *KRT4*, *RPS2*, *KRT6A*, *ACTG2*, *A2ML1*, *EEF1A1*, *RPS8*, *FTH1*, *RPLP0*) (Figure 3E, Supplementary Table S10).

### 3.5. Expression patterns of DEmRNAs in omasum tissue

**Figure 4:**
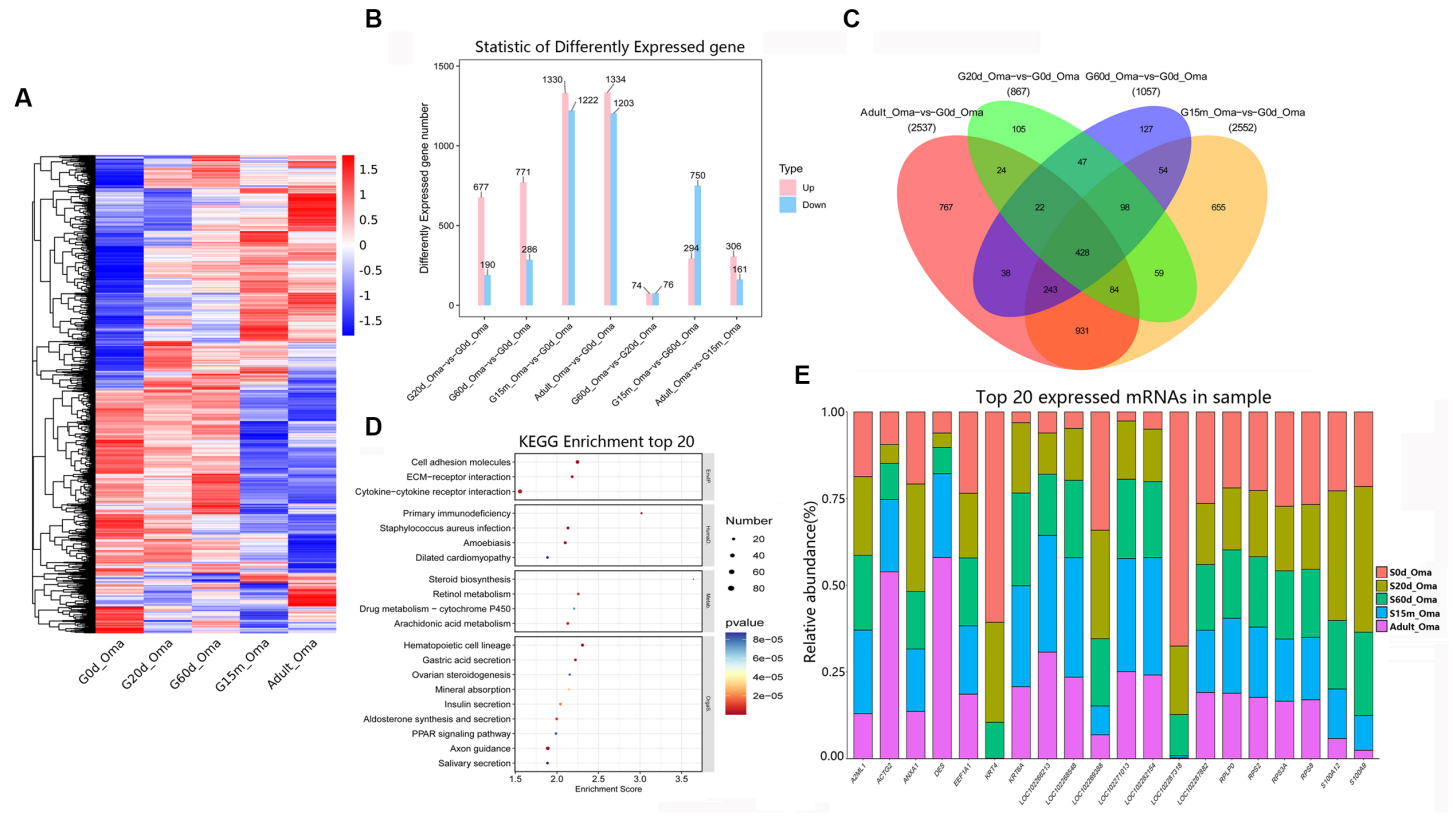
Expression patterns of DEmRNAs in omasum. **(A)** Heatmap of all DEmRNAs among the seven compared groups. **(B)** The number of DEmRNAs. **(C)** The number of common DEmRNAs. **(D)** KEGG pathway analysis of common DEmRNAs. The top 20 enriched KEGG pathways ranked by *p*-values are shown. **(E)** The top 20 expressed mRNAs.

The omasum is a compact spherical organ which composed of the omasal canal and omasal body, but its function is not fully known. A total of 3,975 DEmRNAs were identified among these comparison groups (Figure 4A). In four closed groups, compared with 0 d, 677, 771, 1,330, and 1,334 DEmRNAs were up-regulated at 20 d, 60 d, 15 m, and adult (Figure 4B), and the number of down-regulated DEmRNAs was 190, 286, 1,222, and 1,203, respectively. The number of upregulated DEmRNAs was 74, 294, and 306 in three consecutive groups, while the downregulated DEmRNAs were 76, 750, and 161, respectively (Figure 4B).

During the entire development stages, compared with 0 d, 428 DEmRNAs were considered as common DEmRNAs, revealing that the KEGG pathways were significantly enriched in the cell adhesion molecules, ECM-receptor interaction, cytokine-cytokine receptor interaction, primary immunodeficiency, *staphylococcus aureus* infection, steroid biosynthesis, retinol metabolism, arachidonic acid metabolism, hematopoietic cell lineage, and gastric acid secretion, etc. (Figures 4C,D, Supplementary Table S11). Notably, among the most abundant mRNAs expressed in the omasum, several mRNAs mainly were related to the key roles of omasum developmentand immune response (e.g., *KRT4*, *S100A12*, *S100A9*, *RPS2*, *KRT6A*, *A2ML1*, *EEF1A1*, *RPS8*, *ACTG2*, *ANXA1*) (Figure 4E, Supplementary Table S12).

### 3.6. Expression patterns of DEmRNAs in abomasum tissue

**Figure 5:**
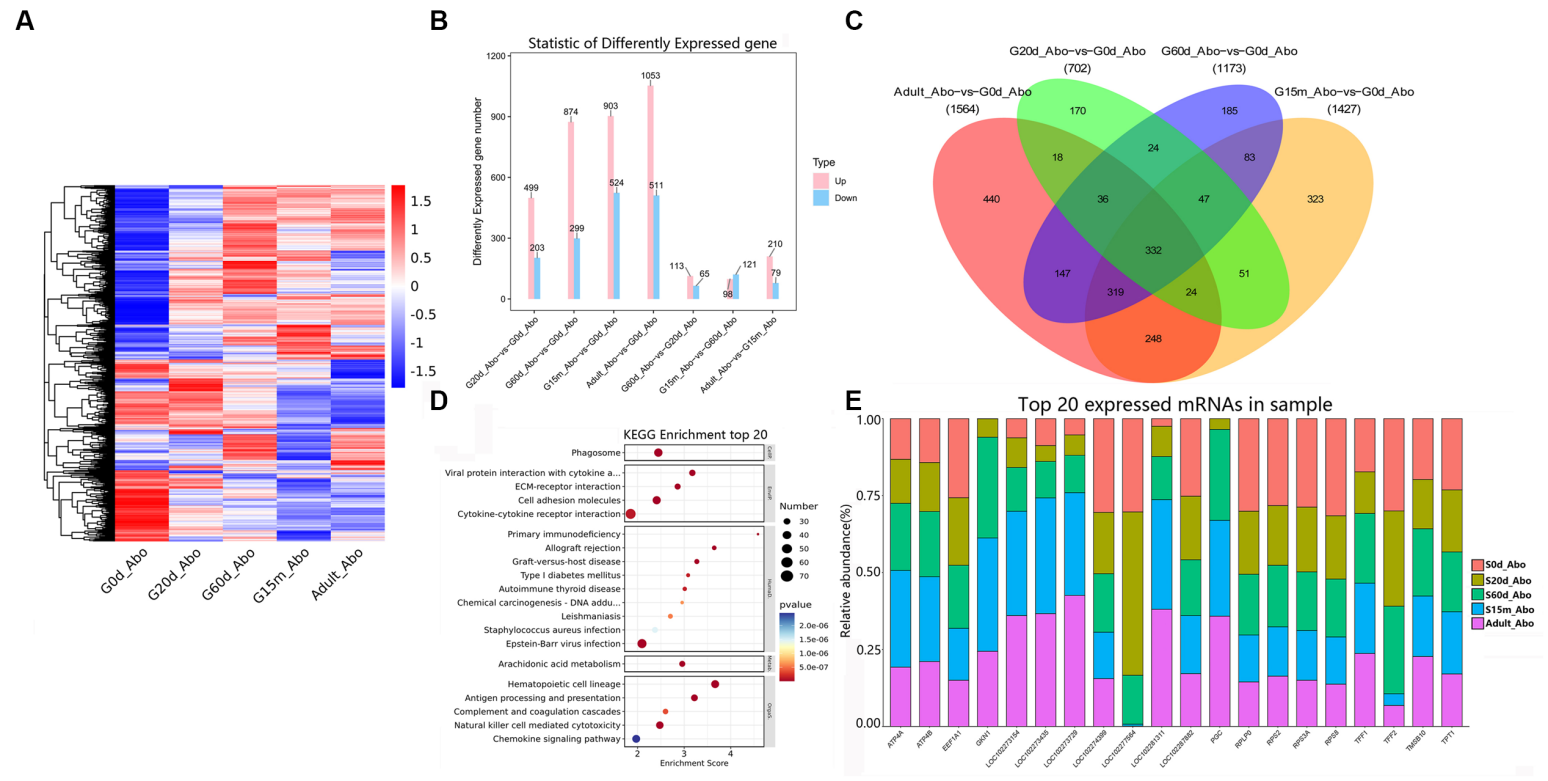
Expression patterns of DEmRNAs in abomasum. **(A)** Heatmap of all DEmRNAs among the seven groups. **(B)** The number of DEmRNAs. **(C)** The number of common DEmRNAs. **(D)** KEGG pathway analysis of common DEmRNAs. The top 20 enriched KEGG pathways ranked by *p*-values are shown. **(E)** The top 20 expressed mRNAs.

Abomasum is the real “stomach,” which can secrete digestive enzymes. In addition to the digestive function of protein in feed, the gastric juice of abomasum can also kill the microorganisms in chyme and provide nutrition for ruminants. Among the seven comparison groups, we clustered 2,638 identified DEmRNAs to obtain an overview of DEmRNAs in the abomasum (Figure 5A). We detected 499, 874, 903, and 1,053 upregulated DEmRNAs at 20 d, 60 d, 15 m and adult in four closed time points (Figure 5B). At these four closed time points, compared with 0 d, KEGG analysis showed that the up-regulated pathways were remarkably enriched in the immune-related pathways, including cell adhesion molecules, hematopoietic cell lineage, natural killer cell-mediated cytotoxicity, Th17 cell differentiation, and Th1 and Th2 cell differentiation, etc. However, in two comparison groups (20 d vs. 0 d and 60 d vs. 0 d), the down-regulated KEGG pathways were primarily related to such protein metabolism and disease-related metabolic pathways as protein processing in endoplasmic reticulum, parkinson disease, ribosome biogenesis in eukaryotes, prion disease, chemical carcinogenesis-DNA adducts and glutathione metabolism, etc. The down-regulated KEGG pathways of the other two closed time points (15 m vs. 0 d and adult vs. 0 d) were all related to relaxin signaling pathway, protein digestion and absorption, amoebiasis, and AGE-RAGE signaling pathway in diabetic complications, and ECM-receptor interaction. Moreover, between 20 d and 60 d, remarkably up-regulated pathways were principally involved in antigen processing and presentation, steroid hormone biosynthesis, DNA replication, natural killer cell mediated cytotoxicity and cell cycle, and down-regulated pathways are mainly related to drug metabolism-cytochrome P450, aldosterone-regulated sodium reabsorption, and cGMP-PKG signaling pathway, etc. Compared to 60 d, the up-regulated pathways at 15 m were primarily enriched in drug metabolism-cytochrome P450, bile secretion, and the down-regulated pathways are primarily related to ECM-receptor interaction, cell cycle, protein digestion and absorption, cAMP signaling pathway, and DNA replication. Compared to 15 m, the up-regulated pathways at adult are primarily related to cell cycle, DNA replication, oocyte meiosis and progesterone-mediated oocyte maturation, and down-regulated pathways are primarily related to PPAR signaling pathway, chemical carcinogenesis-DNA adducts, pentose and glucuronate interconversions, retinol metabolism, and steroid hormone biosynthesis (Supplementary Table S13).

During the entire development stages, compared to 0 d, 332 DEmRNAs were identified as common DEmRNAs, indicating that KEGG pathways were primarily involved in the phagosome, viral protein interaction with cytokine and cytokine receptor, ECM-receptor interaction, cell adhesion molecules, primary immunodeficiency, arachidonic acid metabolism, hematopoietic cell lineage, and antigen processing and presentation (Figures 5C,D, Supplementary Table S14). Remarkably, several of the most abundant DEmRNAs in abomasum from protein-coding genes played pivotal roles in abomasum development, gastric acid secretion and digestive enzyme activity (e.g., *TFF1*, *TMSB10*, *GKN1*, *ATP4B*, *RPS2*, *PGC*, *TFF2*, *EEF1A1*, *TPT1*, *ATP4A*, *RPS3A*, *RPLP0*, and *RPS8*) (Figure 5E, Supplementary Table S15).

### 3.7. DEmRNAs in ruminant stomach

**Figure 6:**
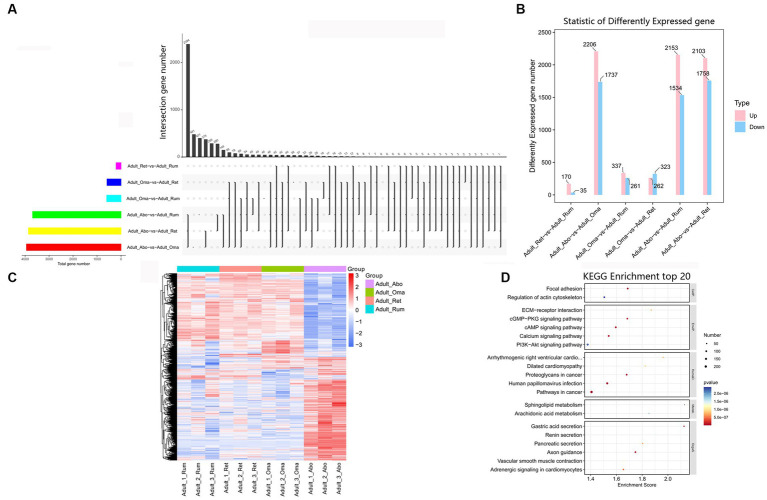
Expression analysis of mRNAs among four stomachs of yak. **(A)** Venn chart of DEmRNAs detected in rumen, reticulum, omasum and abomasum. **(B)** The number of DEmRNAs among four stomachs. **(C)** Heatmap illustrating the relative expression of DEmRNAs from rumen, reticulum, omasum and abomasum tissues at adult. Rows represent mRNAs and columns represent different tissues. **(D)** KEGG pathway analysis of DEmRNAs among four stomachs tissues. The top 20 enriched KEGG pathways ranked by *p*-values are presented.

There are few reports on association analysis of the mRNAs of the four stomachs in yak. To identify the key mRNAs regulating differences in growth and functional metabolism, we evaluated the expression levels and analyzed differential expression of mRNAs in four stomachs (Figure 6A). Compared to the abomasum, we found that 3,687, 3,861, and 3,943 mRNAs were differentially expressed in rumen, reticulum, and omasum, respectively (Figure 6B). In addition, we also found that there were relatively few differential genes among rumen, reticulum and omasum, including 205 DEmRNAs between rumen and reticulum, 598 DEmRNAs between rumen and omasum, and 585 DEmRNAs between reticulum and omasum (Figure 6B). To further clarify the expression pattern of DEmRNAs among four stomachs, hierarchical cluster analysis was performed, and the expression heatmap of DEmRNAs was generated. The results displayed that the expression patterns of these mRNAs were remarkably different between forestomach and abomasum in adult groups (Figure 6C). KEGG enrichment analysis illustrated that these DEmRNAs were principally focused on axon guidance, focal adhesion, gastric acid secretion, cAMP signaling pathway, cGMP-PKG signaling pathway, arachidonic acid metabolism, PI3K-Akt signaling pathway, and regulation of actin cytoskeleton, etc. (Figure 6D, Supplementary Table S16). Therefore, these DEmRNAs were potential candidates for the regulation of development and regeneration, immune response, lipid metabolism and regulation of actin cytoskeleton.

### 3.8. Time-series analysis (STEM) of mRNAs in rumen, reticulum, omasum, and abomasum

**Figure 7:**
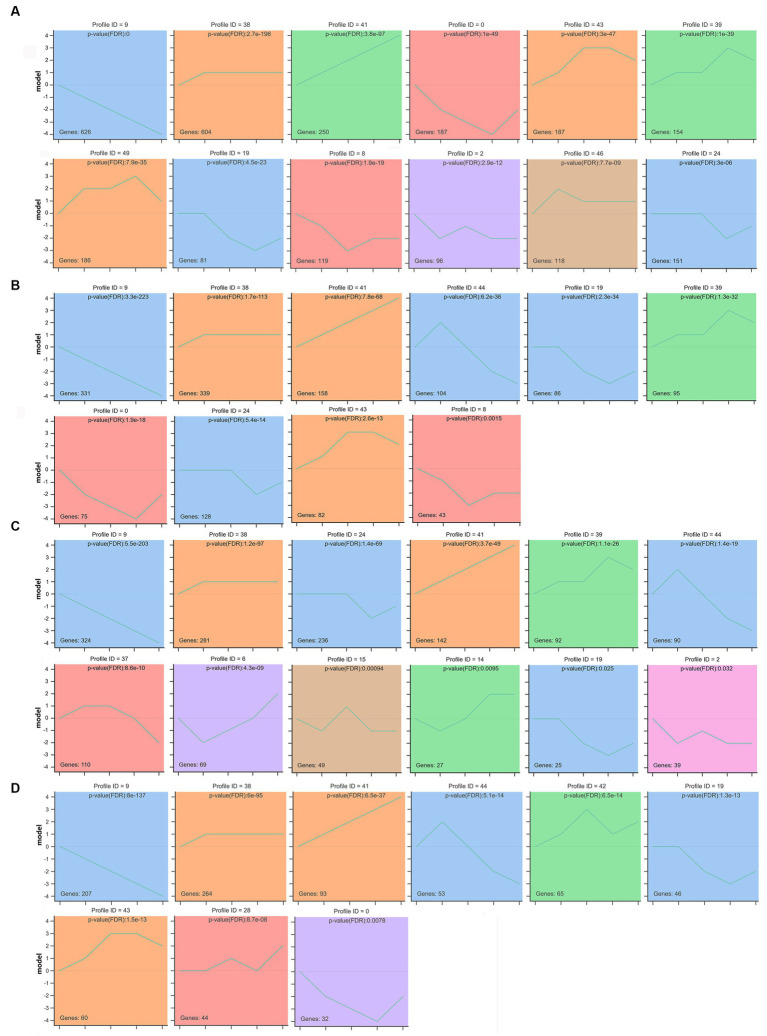
STEM analysis determined temporal expression profiles of mRNAs in rumen **(A)**, reticulum **(B)**, omasum **(C)**, and abomasum **(D)**. The top panel indicates the module number. The number in the upper right corner represents a statistically significant *p*-value. The number at the bottom left shows the number of mRNAs in each profile module. Different colors indicate the colored profiles (Bonferroni-adjusted *p* values < 0.05). The *p*-value is ranked from smallest to largest. Profiles with the same color belong to the same cluster.

According to the dynamic expression patterns of the mRNAs at the five developmental periods, all identified mRNAs in rumen, reticulum, omasum and abomasum were classified into 6, 4, 7, and 5 cluster profiles, containing 12, 10, 12, and 9 significantly enriched model profiles, respectively (Figures 7A–D). Among them, modules 9, 19, 38, and 41 were significantly enriched in rumen, reticulum, omasum and abomasum, and modules 46 and 49 were only significantly enriched in the rumen. Modules 9, 38, and 41 displayed a gradual increase or decrease trend. Modules 2 and 15 showed an “wavy” type expression patterns that were firstly reducing, then gradually increasing, and then decreasing, while modules 28 and 42 showed almost the opposite expression patterns. Modules 37, 39, 43, 44, and 46 showed the expression patterns that gradually increased and then progressively reduced, while modules 0, 6, 8, 14, 19, and 24 showed the opposite expression patterns. Finally, we observed colored modules, and considered the most significant three modules (modules 9, 38, and 41). Depending on the degree of significance, GO enrichment analysis of module profile 9 in four stomachs showed that the genes were mainly enriched for heparin binding, extracellular matrix structural constituent, proteinaceous extracellular matrix, extracellular matrix organization, calcium ion binding, cell adhesion (Supplementary Figures S2A–D). Module 38 showed that 20 days was an important node for regulating immune response, metabolism-related hormone levels, and enzymatic activity in four stomachs tissues (Supplementary Figures S2E–H). Module 41 was participated in fatty acid beta-oxidation, tricarboxylic acid cycle and pyruvate metabolic process (rumen), motor activity, mitochondrial matrix, fatty acid beta-oxidation and non-canonical Wnt signaling pathway (reticulum), negative regulation of canonical Wnt signaling pathway, actin filament, AMPA glutamate receptor complex, and channel regulator activity (omasum), immune response, response to sucrose, phosphatidylinositol-4,5-bisphosphate 3-kinase activity, and transmembrane receptor protein tyrosine kinase signaling pathway (abomasum) (Supplementary Figures S2I–L). In addition, 147, 101, 127, and 93 KEGG pathways of DEmRNAs were enriched in profile 41 in rumen, reticulum, omasum and abomasum, respectively (Supplementary Table S17). In rumen and reticulum, DEmRNAs were involved in valine, leucine and isoleucine degradation, citrate cycle (TCA cycle), propanoate metabolism, pyruvate metabolism, butanoate metabolism and PPAR signaling pathway, and these pathways were closely associated with amino acid metabolism, carbohydrate metabolism and lipid metabolism. In omasum, DEmRNAs participated in the focal adhesion, purine metabolism, ECM-receptor interaction, Wnt signaling pathway and hematopoietic cell lineage, and these pathways were related to cell growth, amino acid metabolism and immune regulation. In abomasum, DEmRNAs were mainly involved in the intestinal immune network for IgA production, cytokine-cytokine receptor interaction, Fc epsilon RI signaling pathway, sphingolipid signaling pathway, adipocytokine signaling pathway, fatty acid biosynthesis, and protein digestion and absorption, and these pathways were closely associated with intestinal immune regulation, protein metabolism and lipid metabolism (Supplementary Table S18).

### 3.9. Co-expression network analysis of mRNAs in rumen, reticulum, omasum, and abomasum

**Figure 8:**
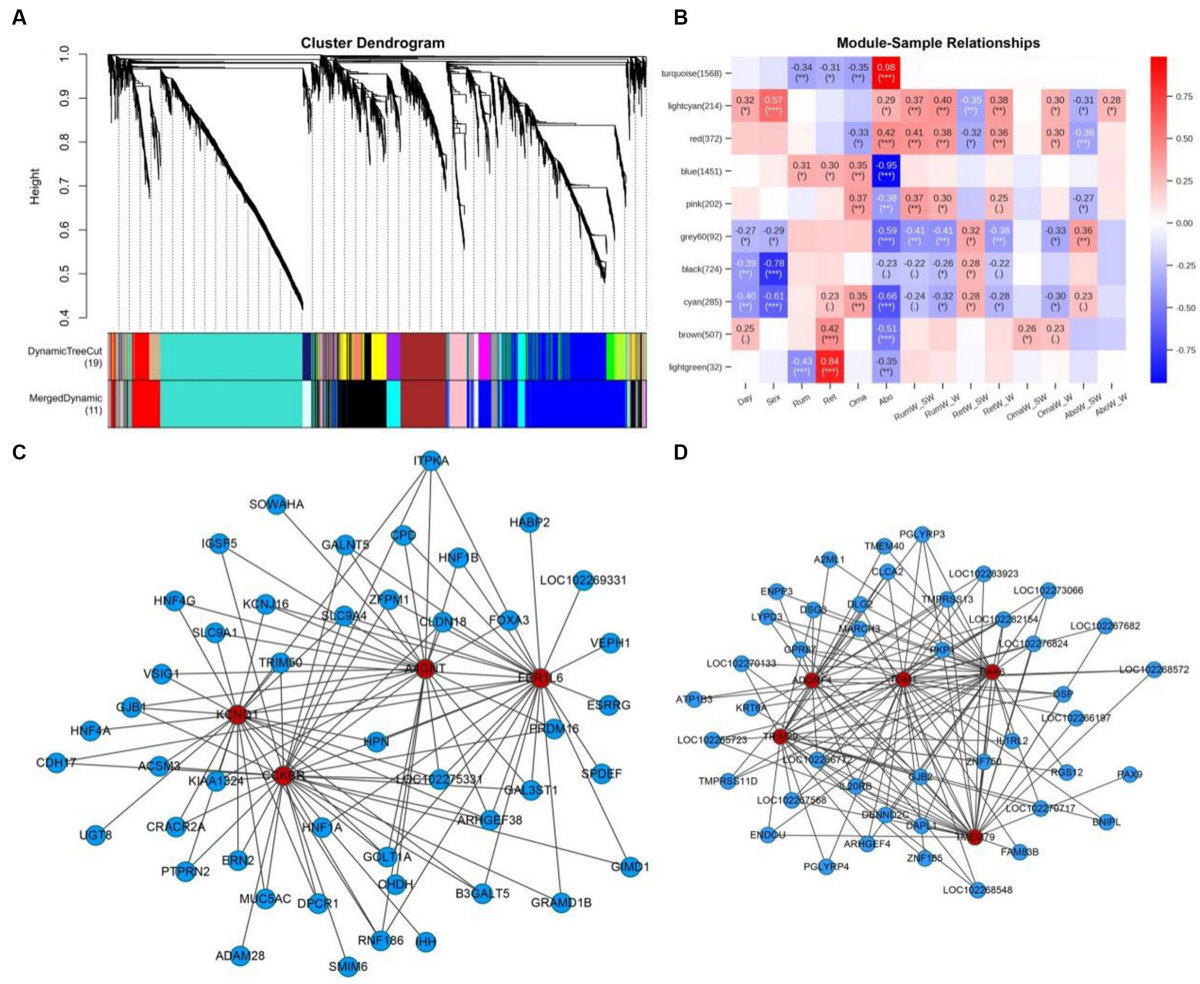
The WGCNA network analysis of 59 samples and the expression profiles of the key modules. **(A)** Hierarchical clustering dendrogram of mRNAs co-expression modules in rumen, reticulum, omasum and abomasum tissues. Each branch shows a cluster of mRNAs. Dynamic tree cut represents original split module, and merged dynamic represents the final merged modules. **(B)** Module-sample correlation heatmap. The number on the left shows the number of module genes. The color scale on the right represents correlation from −1 (blue) to 1 (red). Day represents developmental stages, and Sex represents male and female yaks. RumW_SW represents rumen weight/total weight of four stomachs, and RumW_W represents rumen weight/body weight. **(C)** The co-expression network diagram of mRNAs of four tissues in the turquoise module. **(D)** The co-expression network diagram of mRNAs of four tissues in the blue module. Color indicates different genes, red: hub gene; blue: related genes of hub gene.

A total of 18,575 genes were collected in the original data. After filtering out genes with low expression fluctuation (standard deviation ≤ 0.5), 5,486 genes were eventually left. By WGCNA analysis, a total of 5,486 genes were categorized into 10 modules (the gray module was excluded) (Figure 8A). In module-sample correlation analysis, we found the turquoise and light green modules had the highest correlations with abomasum and reticulum (Figure 8B). We also found that the turquoise and blue modules were sample-specific. The turquoise module was positively correlated with the abomasum, and negatively correlated with rumen, reticulum and omasum. The blue module was the opposite of the turquoise module. Genes in the turquoise (1568) and blue (1451) modules were expressed more highly than the other modules. Among them, *CCKBR*, *KCNQ1*, *FER1L6*, and *A4GNT* were the hub genes of the turquoise module (Figure 8C); *PAK6*, *TRIM29, ADGRF4*, *TGM1*, and *TMEM79* were the hub genes of the blue module (Figure 8D). Through GO enrichment analysis, turquoise and blue modules were principally participated in the biological process (48.31–63.61%), followed by molecular function (22.15–26.69%), and least by cellular components (14.24–25.00%) (Supplementary Tables S19, S20). In the turquoise module, KEGG enrichment analysis indicated that these co-expressed genes were involved in gastric acid secretion, sphingolipid metabolism, ether lipid metabolism, pancreatic secretion, and glycosphingolipid biosynthesis-globo and isoglobo series. In the blue module, the co-expressed genes were participated in pancreatic secretion, riboflavin metabolism, pantothenate and CoA biosynthesis, proximal tubule bicarbonate reclamation, regulation of actin cytoskeleton, and starch and sucrose metabolism (Supplementary Tables S21, S22).

### 3.10. RT-qPCR quantification of mRNAs

Sixteen mRNAs—*S100A12*, *KRT4*, *KRT6A*, *ACTG2*, *A2ML1* (rumen)—*S100A9*, *ACTG2*, *FTH1*, *DES*, *RPLP0* (reticulum)—*ANXA1*, *RPS3A*, *RPLP0*, *A2ML1*, *KRT4* (omasum)—*TFF1*, *GKN1*, *ATP4B*, *PGC*, *RPS8* (abomasum)—were randomly selected for reliable verification of RNA-seq data in yak. The selected genes were quantitatively analyzed by RT-qPCR at five developmental periods. The results presented that RT-qPCR data was consistent with RNA-seq data, revealing that RNA-seq data was dependable (Supplementary Figures S3–S6).

## 4. Discussion

The development of ruminant stomach of yak is closely related to its health and performance, which has attracted wide attention (11, 12, 15). However, the development mechanism of the whole ruminant stomach remains unclear (16), and further studies are needed to provide theoretical references and new perspectives for production in yak. In this study, 3,240, 3,968, 3,975, and 2,638 DEmRNAs were identified in rumen, reticulum, omasum and abomasum, respectively. In these heatmaps, the DEmRNAs expression patterns at 0 d were significantly different from those at the other four stages. These results coincide with those of others, which found that the gene expression patterns of rumen at birth were completely different from those in youth and adulthood in Simmental beef cattle (34). We also found that the DEmRNAs expression patterns at 20 d and 60 d were similar, as well as at 15 m and in adulthood in four stomachs. This is consistent with the rumen status observed at different development stages during dissection. In rumen, we found no solid contents at 0 d, very little pasture and a lot of milk at 20 d, some pasture and a lot of milk at 60 d, and only pasture in the 15 m and adult groups. Indeed, during the suckling and weaning periods, dietary structure changes may also influence the digestive function. This may be related to DEmRNAs (9, 12). Nevertheless, nearly half of the DEmRNAs were down-regulated at 15 m and adult, indicating that the regulatory roles of these DEmRNAs at these stages were relatively poorer than at other three stages in four stomachs. After 15 months, yaks are almost sexually mature and has a mature body with relatively complete organ, which may be the reason why these DEmRNAs are down-regulated.

In this study, we constructed venn diagrams to classify the common DEmRNAs along five developmental stages in rumen, reticulum, omasum and abomasum. In the common DEmRNAs of rumen, we found that *HMGCS2*, *PPARG*, *ACADS*, *ECHS1*, *CBR4*, *ABHD6*, *AKR1A1*, and *INSIG1* were involved in lipid metabolism, *CCNB1*, *KRT6A*, *IGF2BP2*, *E2F8*, *CORO2B*, *RPS6KA3*, *CDKN2C*, *CRISPLD2*, *CREB3L1*, *FSTL3*, *FZD5*, and *CKAP2L* were related to cell cycle, cell differentiation and proliferation, and *CX3CR1*, *CD1E*, *ARID5A*, *C3AR1*, *CD3D*, *CHGA*, *FCER2*, *IL1R2*, *IL36G*, and *IL27RA* were participated in immunoregulatory and inflammatory processes. HMGCS2, a rate-limiting enzyme that regulates ketone body synthesis, acts an important role in the VFA metabolism of the rumen epithelium (35–37). ACADS, a member of the acyl-CoA dehydrogenase family, catalyzes the initial step of the mitochondrial fatty acid β-oxidation pathway. Mutation of this gene can cause SCAD deficiency an acute acidosis and muscle weakness in infants and lipid-storage myopathy in adults (38, 39). CCNB1, also known as cyclin B1, is involved in the cell division and cell cycle. CCNB1 also plays critical roles in accelerating cell cycle and rumen development in goats (40). CX3CR1, C-X3-C motif chemokine receptor 1, is the only receptor for the chemokine fractalkine (CX3CL1). CX3CL1 belongs to the CX3C subgroup of chemokines and mediates the chemotaxis and adhesion of inflammatory cells through CX3CR1 (41). In the present study, CX3CR1 were observed in four closed groups, further indicating that changes in dietary structure may trigger the persistence of rumen epithelial local inflammation throughout the development stage. Interestingly, these genes associated with fatty acid metabolism, such as *HMGCS2*, *PPARG*, and *ACADS* could also be observed in the common DEmRNAs of reticulum and omasum, but not in abomasum. In addition, according to the correlation analysis of the four stomachs, the expression pattern of abomasum was remarkably different from that of rumen, reticulum and omasum. This also indicated that the four stomachs, especially the rumen and abomasum, had different developmental pathways after birth and subsequent onset of rumination.

From the KEGG pathway analysis of common DEmRNAs in abomasum, among the most abundant mRNAs expressed in abomasum, several mRNAs (e.g., *TFF1*, *TMSB10*, *GKN1*, *ATP4B*, *RPS2*, *PGC*, *TFF2*, *EEF1A1*, *TPT1*, *ATP4A*, *RPS3A*, *RPLP0*, and *RPS8*) that influence the important roles of abomasum development, gastric acid secretion and digestive enzyme activity are significantly expressed in abomasum. Gastrokine-1 (GKN1), a member of the gastrokine family, is an anti-amyloid secreted by the stomach, has mitogenic activity, actively acts in protecting the integrity of the gastric mucosal epithelium and regulates diet-induced obesity (42). Pepsinogen C (PGC), a member of the aspartic protease family, is secreted by gastric chief cells. PGC can be activated by pepsin C to digest peptides and amino acids in the abomasum (43). Ribosome biogenesis in eukaryotes requires the participation of 80 ribosomal proteins, and its rate must be synchronized with cellular growth. *RPS2*, *RPS3A*, *RPLP0,* and *RPS8* each encode a ribosomal protein that regulates the translation and controls cell growth, and may further promote the development of abomasum (44, 45). These findings suggest that these DEmRNAs may be associated with abomasum development, gastric acid secretion and digestive enzyme activity, which may affect the growth of yaks. To further understand the role and regulation of these DEmRNAs, it is necessary to study the functions of the identified DEmRNAs. Better understanding of these DEmRNAs may help producers to manipulate and further improve yak yield.

We realize that most biological processes are dynamic, so sequential experiments (time series) are critical for researchers to gain insight into these processes (46). STEM analysis revealed that mRNAs expression patterns were dynamic and similar during the development of the four stomachs in yaks. In rumen, the STEM analysis showed that mRNAs profiles 39 and 41 belonged to the same cluster enriched in amino acid metabolism and lipid metabolism. In profile 41, short-chain enoyl-CoA hydratase, short chain 1 (ECHS1) was involved in amino and fatty acid catabolism in mitochondria, mainly catalyzing the hydration step of fatty acids, and its deficiency can lead to Leigh syndrome or exercise-induced dystonia (47, 48). *ECHS1* had higher expression levels at 20 d, 60 d, 15 m and adult compared to 0 d. The Acyl Coenzyme A oxidase 2 (ACOX2), a member of the acyl-CoA oxidase family, primarily participated in the degradation of long-branched fatty acids and bile acid intermediates in peroxisomes. Lack of this enzyme leads to the accumulation of branched fatty acids and bile acid intermediates (49). Fatty acid beta oxidation is an essential pathway for energy production in growth-stable newborn livestock, and it has been reported that *ACOX2* may also be involved in fatty acid β-oxidation (50). We also found that *HSD17B2*, *ACADSB*, *ECHS1*, *HADH*, *ACSS2*, *ME1*, *ME3*, and *SLC27A2* were involved in the lipid metabolism. Additionally, the KEGG pathway analysis indicated that these genes principally participated to lipid-related pathways, such as steroid hormone biosynthesis, fatty acid degradation pyruvate metabolism and PPAR signaling pathway.

In abomasum, STEM analysis showed that mRNA profiles 38, 41, and 43 belonged to the same cluster, which was mainly involved in immune regulation, signaling molecules and interaction, energy metabolism and lipid metabolism. *FASLG*, also known as *CD95L*, had higher expression levels at 20 d, 60 d, 15 m and adult compared to 0 d after birth. This may be due to the hypoplasia of the gastrointestinal tract, lower immunity and lower expression level of related genes in newborn calves (0 d). *FASLG* has been reported to be critical in triggering apoptosis of some types of cells such as lymphocytes and defects in this gene may be associated with lymphoproliferation and thus a predisposition to autoimmunity (51). We also found that *ITK*, *ITGAL*, *PIK3CD*, *SH2D1A*, *CXCR3 AICDA*, *IL15*, and *ZAP70* were involved in the regulation of the immune system. KEGG pathway also indicated that these genes were mainly involved in immune-related pathways, such as natural killer cell mediated cytotoxicity, Th17 cell differentiation and intestinal immune network for IgA production.

By the rumen-reticulum-omasum-abomasum-mRNA co-expression network analysis, *CCKBR*, *KCNQ1*, *FER1L6*, and *A4GNT* were determined as the hub genes of turquoise module, while *PAK6*, *TRIM29*, *ADGRF4*, *TGM1*, and *TMEM79* were identified as the hub genes of the blue module. The cholecystokinin B receptor (CCKBR), mainly found in the central nervous system and gastrointestinal tract, has a high affinity for both sulfated and nonsulfated CCK analogs (52). Previous studies have indicated that the genetic inactivation of *CCKBR* causes deficits in the gastrointestinal system, control of food intake, memory and exploration, and anxiety-related behaviors (53–55). However, the functional characterization of *CCKBR* remains scarce in yaks. *A4GNT* encodes α-1,4-N-acetylglucosaminyltransferase, which transfers N-acetylglucosamine (GlcNAc) to core 2 branched O-glycans, forming a unique structure GlcNAcα1-4Galβ-R (56). In addition, *A4GNT* plays a vital role in the biosynthesis of type III mucins. Furthermore, the knockdown of *A4GNT* significantly influences gastric development, gradually leading to gastric dysplasia, precancerous lesion of gastric cancer, dysplasia (57, 58). Since *A4GNT* is a hub gene in the co-expression analysis, it is speculated that *A4GNT* may play an essential regulatory role in maintaining normal metabolic homeostasis in yak stomachs. The p21 protein (Cdc42/Rac)-activated kinase 6 (PAK6) regulates a variety of biological activities, including cell proliferation, apoptosis, invasion, metastasis, cytoskeleton rearrangement and the MAP kinase signaling pathway. It has been found that PAK6 interacts with androgen receptor (AR) and estrogen receptor (ER), two steroid hormone-dependent transcription factors that play important roles in sexual differentiation and development (59). However, the functional characterization of PAK6 remains rare in yak stomachs. The functional and regulatory role of PAK6 in the growth and development of yak stomachs will be further studied.

In addition to hub genes, the gastric acid secretion, hedgehog (Hh) signaling pathway, sphingolipid metabolism, glycine, serine and threonine metabolism, carbohydrate digestion and absorption, mineral absorption, thyroid hormone synthesis and cAMP signaling pathway were also enriched and involved in the development and metabolism of yak stomachs. Hh signaling pathway, a highly conserved signaling pathway, participated in regulating the invertebrate and vertebrate organogenesis and tissue homeostasis such as the gastrointestinal tract (60, 61). Previous studies reveal that Hh signaling may inhibit fat formation and influence the adipogenic differentiation of preadipocytes (62, 63). Hedgehog signaling pathway has also been declared to promote lipolysis in adipose tissue by directly regulating brummer (Bmm)/adipose triglyceride lipase (ATGL) (64). In this study, abundant genes were enriched in the Hh signaling pathway. Consequently, it is speculated that the Hh signaling pathway plays a key role in regulating the lipid metabolism in ruminant stomach, which may provide a reference for improving fat ratio and weight gain of yaks. Thyroid hormone (TH) is an endocrine messenger that regulates metabolic processes necessary for normal growth, development, and function in almost all vertebrates (65–67). It is well determined that TH status is closely related to body weight and energy expenditure (68–70). Previous studies have reported that TH can stimulate both lipogenesis and lipolysis, as well as excess thyroid hormones, promoting a hypermetabolic state characterized by increased resting energy expenditure, elevated cholesterol levels, reduced lipolysis, and gluconeogenesis, etc. (71, 72). Clinically, thyroid dysfunction manifests as dominant or subclinical hypothyroidism that negatively affects lipid metabolism and leads to hypercholesterolemia, which gradually increases the risk of cardiovascular disease and potential mortality (73). The role of TH in energy metabolism and lipid metabolism may be helpful to improve the survival condition of yaks in low oxygen, high cold and high altitude weather. cAMP signaling pathway, a classic pathway, participated in the regulation of key physiological processes, including gene transcription, cell fate, muscle contraction, metabolism, secretion and calcium homeostasis (74–76). cAMP signaling pathway has been declared to control adipocyte differentiation and regulate lipid metabolism in white adipose tissue (77). Previous studies have shown that cAMP signaling pathway can regulate the feed efficiency of yorkshire pigs by participating in the influence of lipid metabolism in adipose tissue. These results show that the hedgehog signaling pathway, thyroid hormone synthesis and cAMP signaling pathway may play a crucial role in maintaining normal homeostasis and growth metabolism in yaks. The ruminant stomachs are the major digestive organs participated in nutrient digestion and absorption in yaks. Living at high altitudes and in cold environments all year round, yaks require normal homeostasis to maintain proper growth. Hence, a deeper understanding of mRNAs co-expression in rumen, reticulum, omasum and abomasum can provide some clues and basic knowledge for improving homeostasis, feed utilization and meat quality, and thus improve the growth and survival of yaks.

## 5. Conclusion

In summary, we first revealed the expression profile of mRNAs in the rumen, reticulum, omasum and abomasum of yaks in five developmental stages. In this study, 3,240, 3,968, 3,975, and 2,638 DEmRNAs were identified in rumen, reticulum, omasum and abomasum, respectively. Overall, the expression patterns of DEmRNAs were unique at 0 d, similar at 20 d and 60 d, and similar at 15 m and adult in four stomachs. 20 days is an important node for regulating immune response, metabolism-related hormone levels, and enzymatic activity in four stomachs tissues. On the whole, the expression pattern of abomasum was greatly different from that of rumen, reticulum and omasum. STEM and WGCNA analysis demonstrated that many important mRNAs were involved in lipids, protein metabolism, saccharides, and other biological processes. They may perform key functions in health, growth and development, and tissue repair of ruminant stomach. The potential regulatory relationship of mRNAs between the four stomachs has been further explored and confirmed. These findings can provide data reference for further study of the function and mechanisms of important mRNAs in the four stomachs of yaks. It also provided an important theoretical basis for the research of ruminant stomach in other yak breeds. This study provides a molecular basis for further research on age-appropriate weaning and supplementary feeding of yak calves, and also provides an important reference for the research on nutrition and feeding management of yaks. This will help improve our understanding of ruminant physiology and facilitate further development of animal husbandry, while reducing overgrazing pressures on the natural grasslands on the Qinghai-Tibetan Plateau.

## Data availability statement

The datasets presented in this study can be found in online repositories. The names of the repository/repositories and accession number(s) can be found below: https://www.ncbi.nlm.nih.gov/geo/query/acc.cgi?acc=GSE222396.

## Ethics statement

The animal studies were approved by The Institutional Animal Care and Use Committee at Southwest Minzu University. The studies were conducted in accordance with the local legislation and institutional requirements. Written informed consent was obtained from the owners for the participation of their animals in this study.

## Author contributions

YL performed data analysis and drafted the manuscript. QM conducted the experiments and data analysis, with equal contributions. JT and LY participated in the sorting of the test data. TP and XM participated in partial operation of the RT-PCR experiments. MJ designed this study and revised the manuscript. All authors contributed to the article and approved the submitted version.

## Funding

This work was supported by the Natural Science Foundation of Sichuan Province (2022NSFSC1665), Southwest Minzu University Research Startup Funds (RQD2022048), Sichuan Science and Technology Program (2023YFQ0076), Sichuan Science and Technology Program (2021YFYZ0001 and 2021YFN0001).

## Conflict of interest

The authors declare that the research was conducted in the absence of any commercial or financial relationships that could be construed as a potential conflict of interest.

## Publisher’s note

All claims expressed in this article are solely those of the authors and do not necessarily represent those of their affiliated organizations, or those of the publisher, the editors and the reviewers. Any product that may be evaluated in this article, or claim that may be made by its manufacturer, is not guaranteed or endorsed by the publisher.

## Footnotes

^1^ http://ccb.jhu.edu/software/hisat2

^2^ http://samtools.sourceforge.net/mpileup.shtml
