# Supplementary material for: Transcriptome profiling in rumen, reticulum, omasum, and abomasum tissues during the developmental transition of pre-ruminant to the ruminant in yaks
Source: Front Vet Sci. 2023 Sep 22;10:1204706. doi: 10.3389/fvets.2023.1204706 (PMC10556492; doi:10.3389/fvets.2023.1204706)
Supplement: Supplementary file 1 [file Data_Sheet_1.zip › Supplemental Materials-0826/Legends of Figure S1-S6.docx]

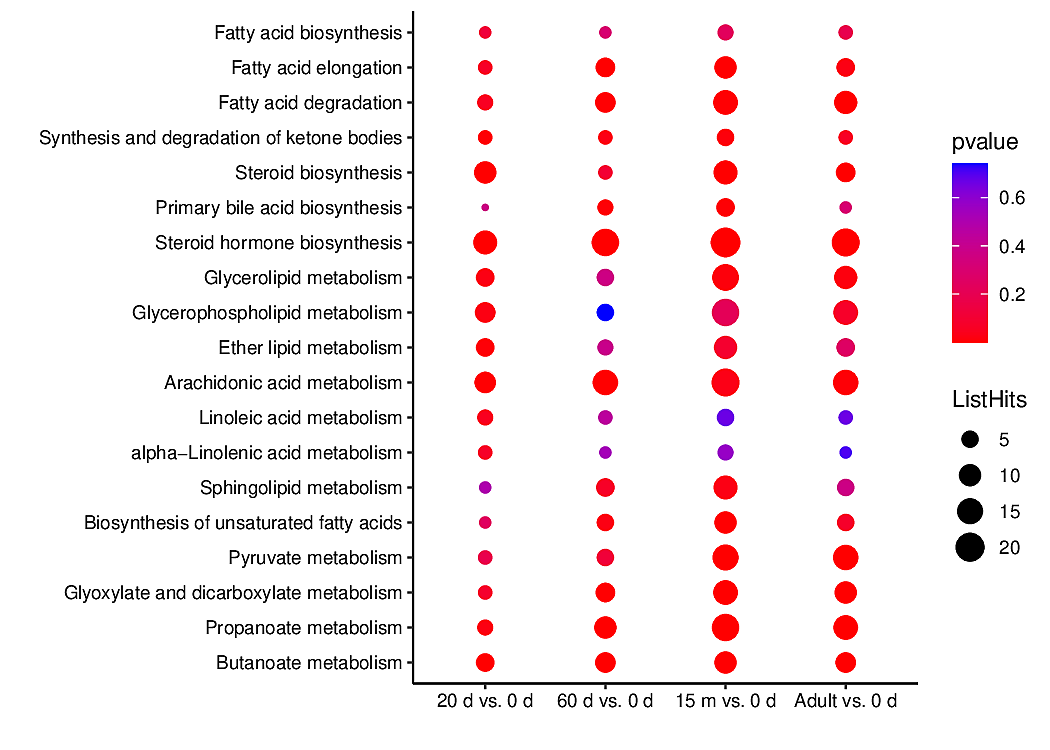
**Figure S1.** KEGG enrichment bubble plot of DEmRNAs involved in fatty acid metabolism in four closed groups.


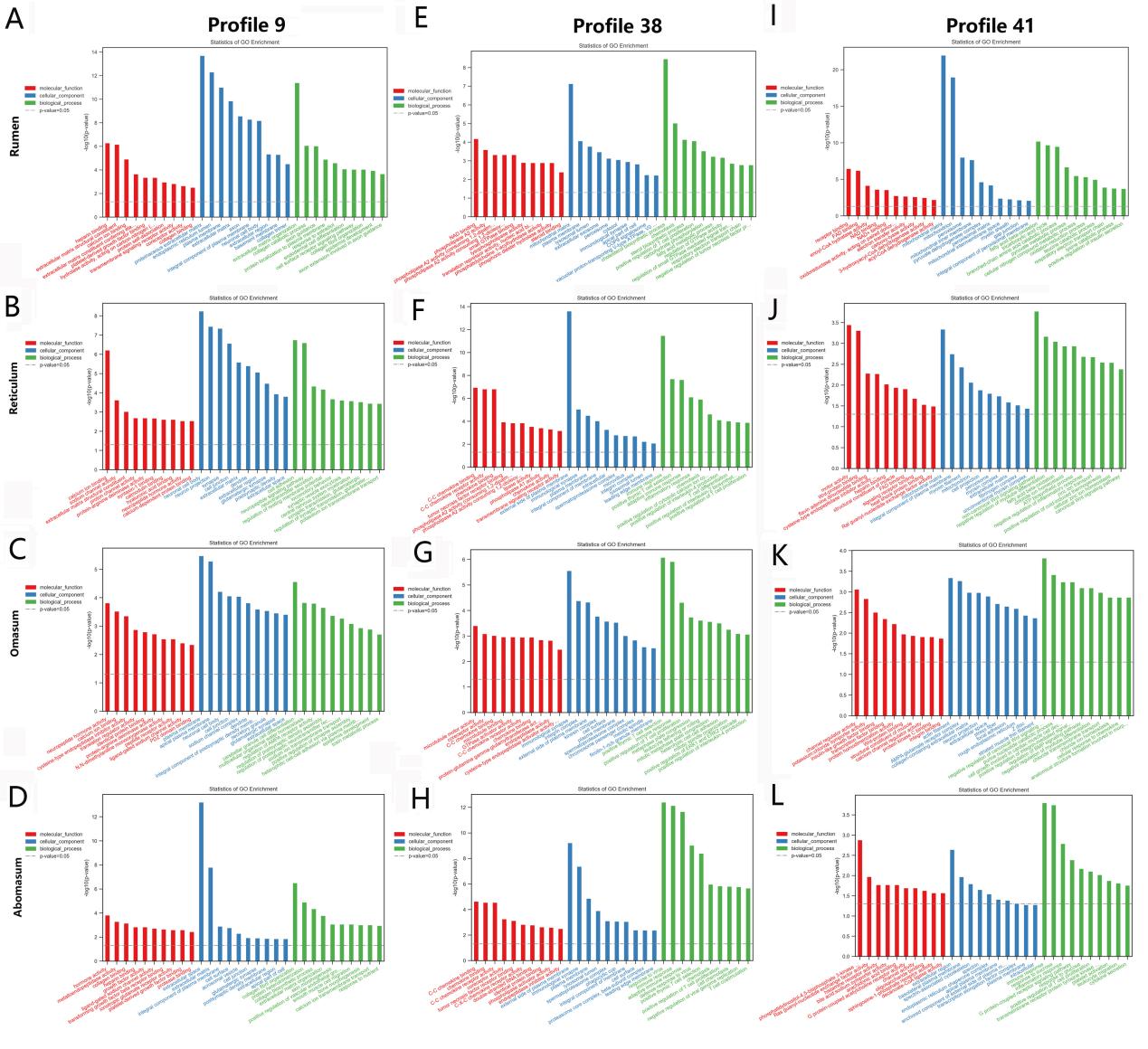


**Figure S2**. The top30 GO enrichment of profile 9, 38 and 41 in four stomachs.


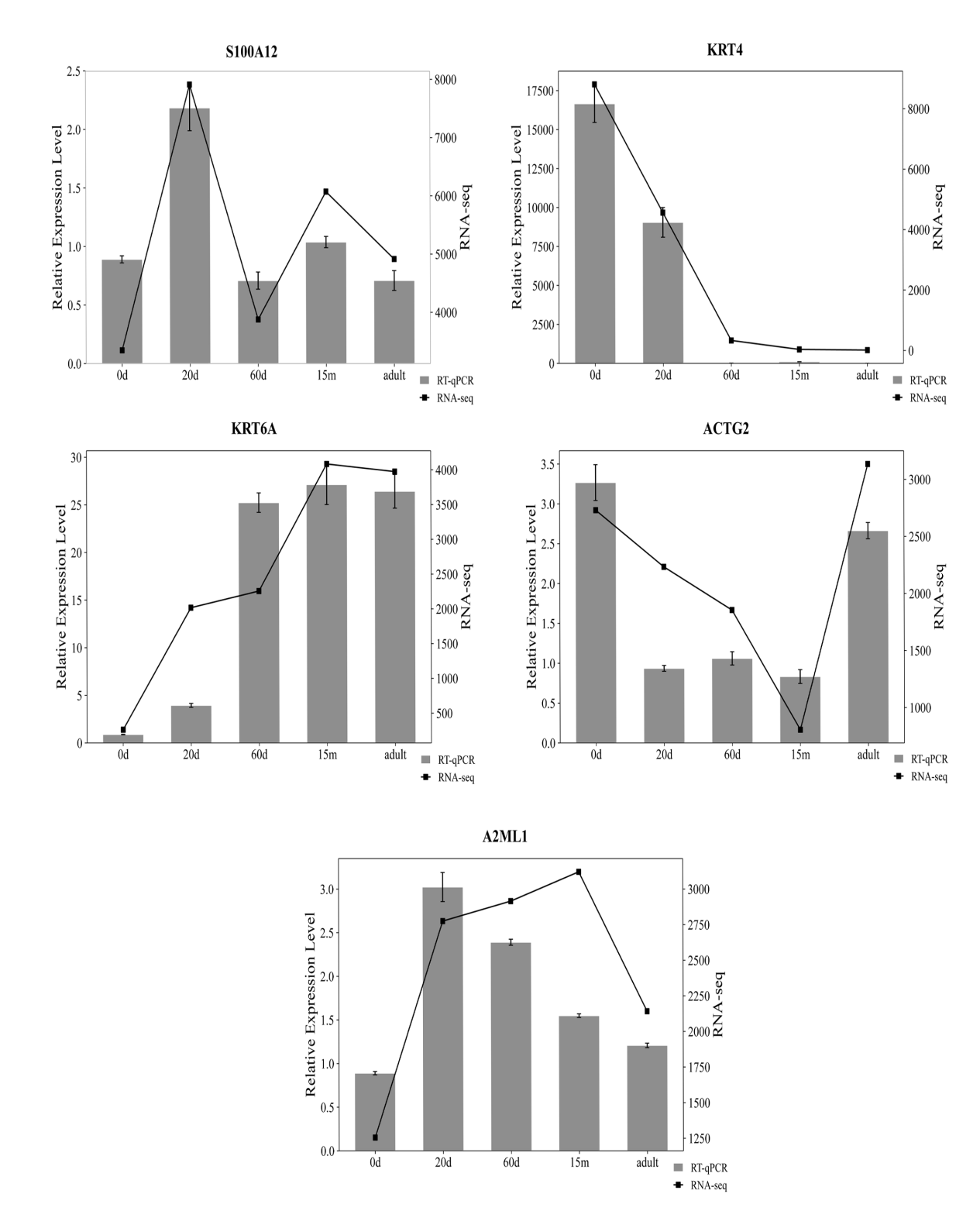


**Figure S3**. Transcription patterns of *S100A12, KRT4, KRT6A, ACTG2, A2ML1* compared to expression patterns in the RNA-seq in rumen.


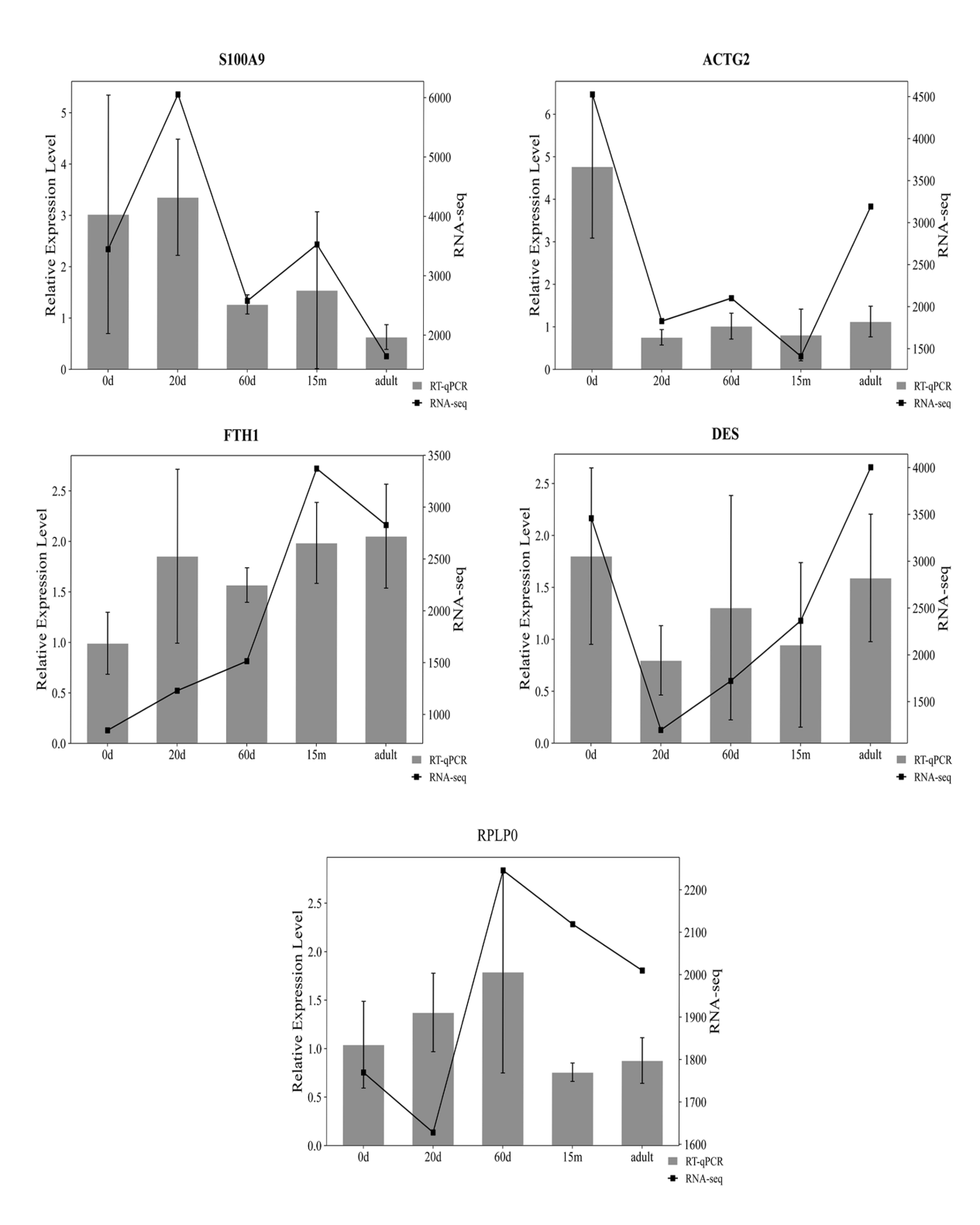
**Figure S4**. Transcription patterns of *S100A9*, *ACTG2, FTH1, DES, RPLP0* compared to expression patterns in the RNA-seq in reticulum.


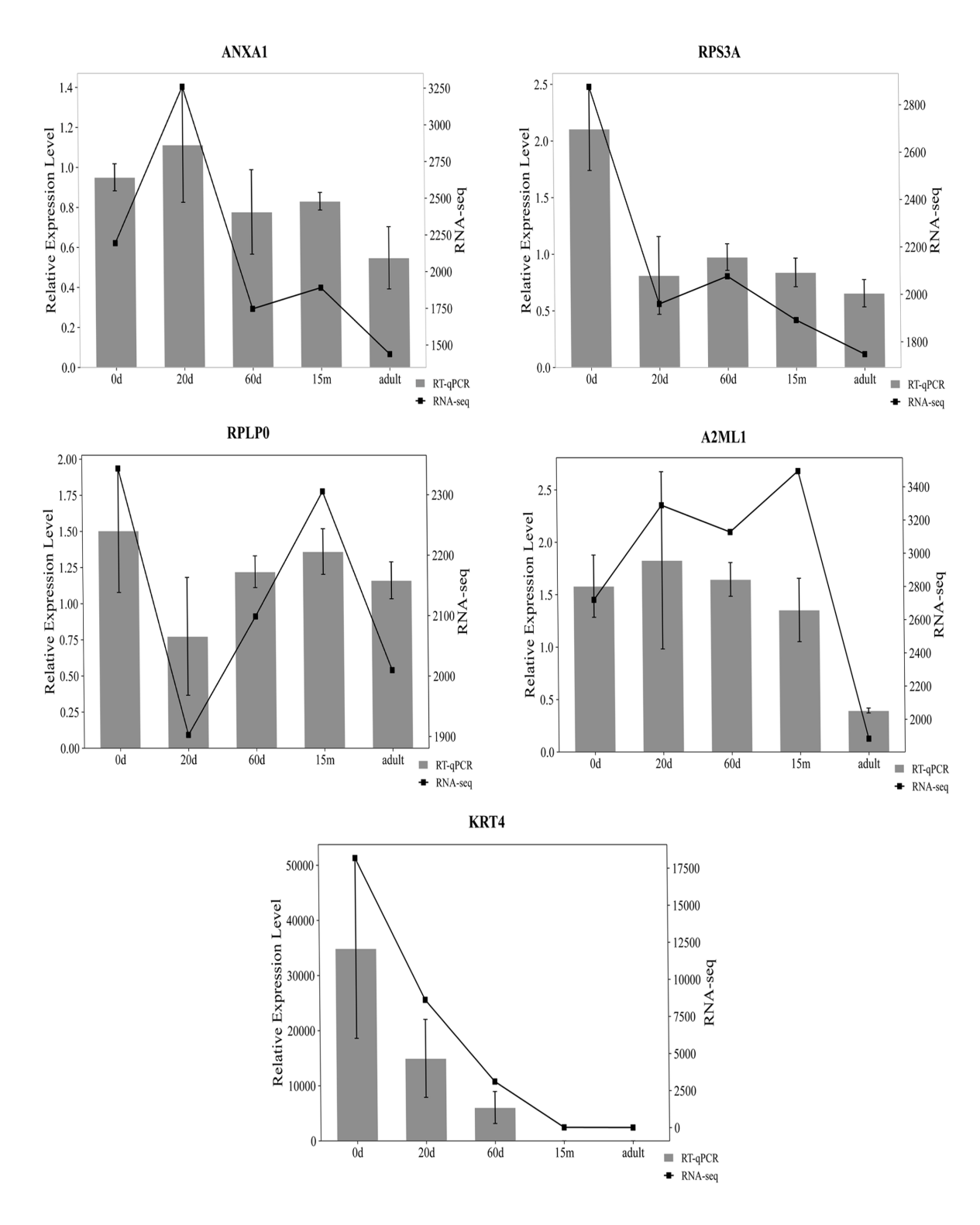
**Figure S5**. Transcription patterns of *ANXA1, RPS3A, RPLP0, A2ML1, KRT4* compared to expression patterns in the RNA-seq in omasum.


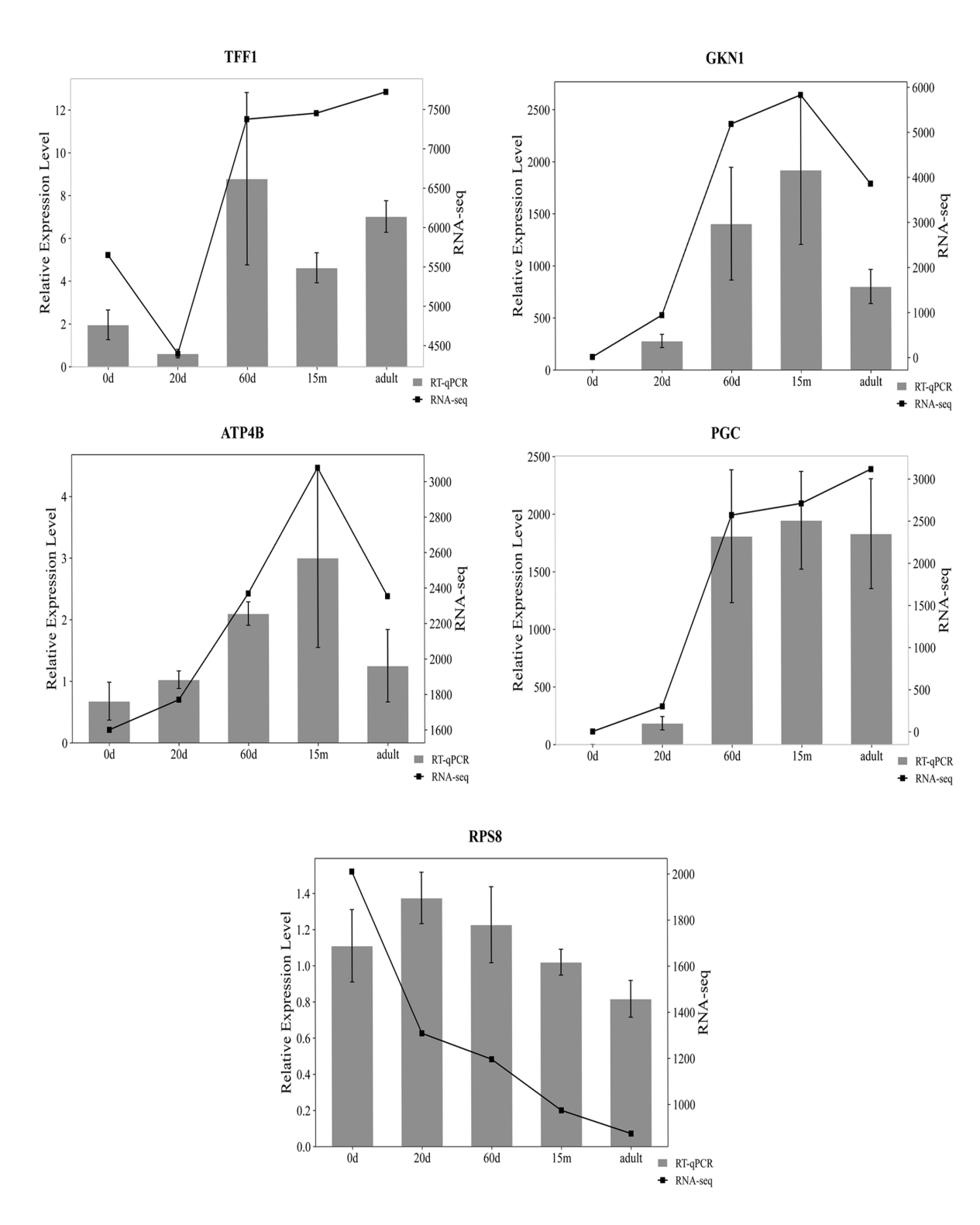
**Figure S6**. Transcription patterns of *TFF1, GKN1, ATP4B, PGC, RPS8* compared to expression patterns in the RNA-seq in abomasum.
