# Supplementary material for: Transcriptome profiling in rumen, reticulum, omasum, and abomasum tissues during the developmental transition of pre-ruminant to the ruminant in yaks
Source: Front Vet Sci. 2023 Sep 22;10:1204706. doi: 10.3389/fvets.2023.1204706 (PMC10556492; doi:10.3389/fvets.2023.1204706)
Supplement: Supplementary file 1 [file Data_Sheet_1.zip › Supplemental Materials-0826/Legends of Table S1-S22.docx]

**Supplementary Information**

**Additional file 1:** Table S1. Forward and reverse primers used for gene quantification by RT-qPCR.

**Additional file 2:** Table S2. Quality analysis of transcriptome sequencing data of ruminant stomach tissues of yaks in five different developmental stages.

**Additional file 3:** Table S3. KEGG enrichment analysis of mRNAs in closed groups (20 d vs. 0 d, 60 d vs. 0 d, 15 m vs. 0 d, and adult vs. 0 d)

**Additional file 4:** Table S4. The common DEmRNAs in rumen.

**Additional file 5:** Table S5. The top 20 KEGG enrichment pathways of common DEmRNAs in rumen.

**Additional file 6:** Table S6. The KEGG enrichment pathways in seven comparison groups in rumen.

**Additional file 7:** Table S7. The top 20 mRNAs expressed in rumen.

**Additional file 8:** Table S8. The KEGG enrichment pathways in seven comparison groups in reticulum.

**Additional file 9:** Table S9. The top 20 KEGG enrichment pathways of common DEmRNAs in reticulum.

**Additional file 10:** Table S10. The top 20 mRNAs expressed in reticulum.

**Additional file 11:** Table S11. The top 20 KEGG enrichment pathways of common DEmRNAs in omasum.

**Additional file 12:** Table S12. Top mRNAs expressed in omasum.

**Additional file 13:** Table S13. The KEGG enrichment pathways in seven comparison groups in abomasum.

**Additional file 14:** Table S14. The top 20 KEGG enrichment pathways of common DEmRNAs in abomasum.

**Additional file 15:** Table S15. The top 20 mRNAs expressed in abomasum.

**Additional file 16:** Table S16. The KEGG pathway analysis of DEmRNAs among four stomachs.

**Additional file 17:** Table S17. The KEGG enrichment in mRNAs module profile 41 of STEM in rumen, reticulum, omasum and abomasum.

**Additional file 18:** Table S18. The top 20 KEGG enrichment in mRNAs module profile 41 of STEM in rumen, reticulum, omasum and abomasum.

**Additional file 19:** Table S19. The GO enrichment in turquoise module of WGCNA.

**Additional file 20:** Table S20. The GO enrichment in blue module of WGCNA.

**Additional file 21:** Table S21. The KEGG enrichment in turquoise module of WGCNA.

**Additional file 22:** Table S22. The KEGG enrichment in blue module of WGCNA.
