# Supplementary material for: Transcriptome profiling in rumen, reticulum, omasum, and abomasum tissues during the developmental transition of pre-ruminant to the ruminant in yaks
Source: Front Vet Sci. 2023 Sep 22;10:1204706. doi: 10.3389/fvets.2023.1204706 (PMC10556492; doi:10.3389/fvets.2023.1204706)
Supplement: Supplementary file 1 [file Data_Sheet_1.zip › Supplemental Materials-0826/Table S1-S22/Table S1-Forward and reverse primers used for gene quantifification by RT-qPCR.docx]

**Table S1** Forward and reverse primers used for gene quantification by RT-qPCR

| **Gene** | **GenBank** | **Primer sequence(5’-3’)** | **Amplicon Size (bp)** |
| --- | --- | --- | --- |
| *S100A12* | XM_014482885.1 | F：TCTTAGCCTCTTAACAGGAAGC | 143 |
|  |  | R：AACTACGTTCACCCCTCTGC |  |
| *KRT4* | XM_005907003.2 | F：CAAGGTCCAGCAGCTTCAGA | 142 |
|  |  | R：CAGAGTCTGGCACTGCTTCT |  |
| *KRT6A* | XM_014481915.1 | F：TGGATTTGGTTTTGGTGGCTTC | 126 |
|  |  | R：TGGATGGTGGGGTCGATTTG |  |
| *ACTG2* | XM_005897639.2 | F：CGTCAGCTGGCCTTTTTAGG | 139 |
|  |  | R：GCAGCTAGGGAACGAGTGTA |  |
| *A2ML1* | XM_014479039.1 | F：TCTTCCACTCAGGACACCGT | 100 |
|  |  | R：CGGATTTCACTGCCAGGTTG |  |
| *S100A9* | XM_014482883.1 | F：TGCCAAACTTTCTCAAGAAGCAG | 149 |
|  |  | R：CATCTCCTCGTGGGAGGCTA |  |
| *FTH1* | XM_005901290.1 | F：AGACCGTGATGACTGGGAGA | 136 |
|  |  | R：AATGAAATCACACAGATGGGGA |  |
| *DES* | XM_005889691.2 | F：CAATTTGGCTGCCTTCCGAG | 134 |
|  |  | R：GTAGCTCGCGGATCTCCTCT |  |
| *RPLP0* | XM_005891475.2 | F：AGCACCGTGTGAAGTCACTG | 132 |
|  |  | R：AGCTGCACATCACTCAGGATT |  |
| *ANXA1* | XM_005887846.2 | F：AGGCCTTGCACAAAGCAATC | 139 |
|  |  | R：ACTTCATCCAGGGGCTTTCC |  |
| *RPS3A* | XM_005902368.2 | F：CAAGAAGAAAGTGGTTGACCC | 132 |
|  |  | R：CCATCAGACGCGATTTTGGT |  |
| *TFF1* | XM_014482112.1 | F：TCACGGCTAAGGAGTGCAAA | 108 |
|  |  | R：AAAACTAGCACGCTTCCTCC |  |
| *GKN1* | XM_005893585.2 | F：ATGAACATGGCGTGGCCAAT | 87 |
|  |  | R：TGACAGCAAAGCCACTTCCG |  |
| *ATP4B* | XM_005910524.2 | F：CTCAAATCGCCAGGGGTGAC | 127 |
|  |  | R：CTGCCAGAAAGTGCCACAAG |  |
| *PGC* | XM_005896448.1 | F：ACTCCACCAATGAGCAGACC | 94 |
|  |  | R：TTGATGCCTTGGACCGTCAG |  |
| *RPS8* | XM_005894184.2 | F：TGAGAGGAAAAAGAACGCGA | 82 |
|  |  | R：TGAAGCGATGCATGCAAGAAG |  |
