# Supplementary material for: Transcriptome profiling in rumen, reticulum, omasum, and abomasum tissues during the developmental transition of pre-ruminant to the ruminant in yaks
Source: Front Vet Sci. 2023 Sep 22;10:1204706. doi: 10.3389/fvets.2023.1204706 (PMC10556492; doi:10.3389/fvets.2023.1204706)
Supplement: Supplementary file 1 [file Data_Sheet_1.zip › Supplemental Materials-0826/Table S1-S22/Table S2-Quality analysis of transcriptome sequencing data of ruminant stomach tissues.docx]

**Table S2** Quality analysis of transcriptome sequencing data of ruminant stomach tissues of yaks in five different developmental stages.

| **Groups** | **Sample** | **Raw_reads** | **Raw_bases** | **Clean_reads** | **Clean_bases** | **Valid_bases** | **Q30** | **GC** |
| --- | --- | --- | --- | --- | --- | --- | --- | --- |
| **Rumen** | S0d_1_Rum | 50.67M | 7.60G | 49.56M | 7.17G | 94.36% | 93.88% | 51.23% |
|  | S0d_2_Rum | 51.06M | 7.66G | 50.20M | 7.27G | 94.93% | 94.47% | 51.00% |
|  | S0d_3_Rum | 51.39M | 7.71G | 50.25M | 7.21G | 93.52% | 93.85% | 51.85% |
|  | S20d_1_Rum | 47.74M | 7.16G | 46.63M | 6.75G | 94.21% | 93.73% | 50.95% |
|  | S20d_2_Rum | 43.30M | 6.49G | 42.25M | 6.08G | 93.68% | 93.55% | 51.36% |
|  | S20d_3_Rum | 48.93M | 7.34G | 47.62M | 6.83G | 93.03% | 93.36% | 50.83% |
|  | S60d_1_Rum | 45.30M | 6.80G | 44.21M | 6.36G | 93.61% | 93.49% | 50.64% |
|  | S60d_2_Rum | 49.09M | 7.36G | 47.90M | 6.92G | 94.00% | 93.53% | 50.97% |
|  | S60d_3_Rum | 49.77M | 7.47G | 48.62M | 7.00G | 93.73% | 93.68% | 50.77% |
|  | S15m_1_Rum | 47.41M | 7.11G | 46.35M | 6.65G | 93.55% | 93.93% | 50.25% |
|  | S15m_2_Rum | 49.77M | 7.47G | 48.61M | 7.02G | 94.02% | 93.77% | 50.30% |
|  | S15m_3_Rum | 46.99M | 7.05G | 45.98M | 6.63G | 94.10% | 93.96% | 50.25% |
|  | Adult_1_Rum | 45.88M | 6.88G | 44.92M | 6.50G | 94.42% | 93.97% | 49.90% |
|  | Adult_2_Rum | 47.07M | 7.06G | 45.99M | 6.65G | 94.13% | 93.88% | 50.57% |
|  | Adult_3_Rum | 46.04M | 6.91G | 45.08M | 6.53G | 94.48% | 93.99% | 50.40% |
| **Reticulum** | S0d_1_Ret | 47.65M | 7.15G | 46.97M | 6.72G | 94.07% | 95.51% | 52.36% |
|  | S0d_2_Ret | 49.38M | 7.41G | 48.68M | 6.99G | 94.39% | 95.45% | 51.67% |
|  | S0d_3_Ret | 51.29M | 7.69G | 50.57M | 7.28G | 94.59% | 95.51% | 52.62% |
|  | S20d_1_Ret | 50.05M | 7.51G | 49.39M | 7.09G | 94.39% | 95.50% | 52.57% |
|  | S20d_2_Ret | 47.16M | 7.07G | 46.57M | 6.72G | 94.98% | 95.70% | 52.44% |
|  | S20d_3_Ret | 48.36M | 7.25G | 47.72M | 6.84G | 94.30% | 95.57% | 51.45% |
|  | S60d_1_Ret | 49.63M | 7.44G | 48.97M | 7.06G | 94.90% | 95.56% | 51.60% |
|  | S60d_2_Ret | 47.92M | 7.19G | 47.26M | 6.76G | 94.08% | 95.51% | 51.95% |
|  | S60d_3_Ret | 48.66M | 7.30G | 47.95M | 6.84G | 93.72% | 95.41% | 52.17% |
|  | S15m_1_Ret | 47.16M | 7.07G | 46.50M | 6.66G | 94.19% | 95.47% | 51.84% |
|  | S15m_2_Ret | 47.83M | 7.17G | 47.18M | 6.77G | 94.39% | 95.45% | 51.11% |
|  | S15m_3_Ret | 50.52M | 7.58G | 49.87M | 7.16G | 94.52% | 95.48% | 51.98% |
|  | Adult_1_Ret | 50.66M | 7.60G | 49.99M | 7.21G | 94.86% | 95.46% | 51.17% |
|  | Adult_2_Ret | 48.15M | 7.22G | 47.51M | 6.83G | 94.59% | 95.52% | 51.12% |
|  | Adult_3_Ret | 51.33M | 7.70G | 50.63M | 7.30G | 94.76% | 95.47% | 51.64% |
| **Omasum** | S0d_1_Oma | 49.99M | 7.50G | 49.33M | 7.11G | 94.76% | 95.47% | 52.15% |
|  | S0d_2_Oma | 51.26M | 7.69G | 50.53M | 7.26G | 94.45% | 95.42% | 51.33% |
|  | S0d_3_Oma | 48.88M | 7.33G | 48.22M | 6.90G | 94.13% | 95.48% | 52.03% |
|  | S20d_1_Oma | 49.14M | 7.37G | 48.47M | 6.95G | 94.27% | 95.47% | 51.68% |
|  | S20d_2_Oma | 49.56M | 7.43G | 48.95M | 6.96G | 93.62% | 95.59% | 52.26% |
|  | S20d_3_Oma | 47.11M | 7.07G | 46.51M | 6.68G | 94.51% | 95.60% | 51.75% |
|  | S60d_1_Oma | 50.08M | 7.51G | 49.43M | 7.07G | 94.10% | 95.59% | 51.63% |
|  | S60d_2_Oma | 50.71M | 7.61G | 50.01M | 7.17G | 94.28% | 95.46% | 51.76% |
|  | S60d_3_Oma | 47.48M | 7.12G | 46.87M | 6.73G | 94.45% | 95.56% | 52.02% |
|  | S15m_1_Oma | 49.54M | 7.43G | 48.84M | 7.00G | 94.14% | 95.52% | 51.69% |
|  | S15m_2_Oma | 51.21M | 7.68G | 50.51M | 7.23G | 94.17% | 95.55% | 51.50% |
|  | S15m_3_Oma | 50.40M | 7.56G | 49.71M | 7.13G | 94.28% | 95.52% | 51.46% |
|  | Adult_1_Oma | 50.49M | 7.57G | 49.83M | 7.17G | 94.64% | 95.50% | 51.43% |
|  | Adult_2_Oma | 48.12M | 7.22G | 47.49M | 6.82G | 94.46% | 95.64% | 51.96% |
|  | Adult_3_Oma | 51.21M | 7.68G | 50.52M | 7.23G | 94.12% | 95.60% | 51.75% |
| **Abomasum** | S0d_1_Abo | 49.55M | 7.43G | 48.88M | 7.01G | 94.29% | 95.61% | 50.70% |
|  | S0d_2_Abo | 48.16M | 7.22G | 47.51M | 6.79G | 93.97% | 95.59% | 51.28% |
|  | S0d_3_Abo | 48.15M | 7.22G | 47.46M | 6.77G | 93.71% | 95.60% | 51.90% |
|  | S20d_1_Abo | 47.30M | 7.10G | 46.68M | 6.69G | 94.35% | 95.75% | 51.73% |
|  | S20d_2_Abo | 50.79M | 7.62G | 50.09M | 7.18G | 94.21% | 95.64% | 51.85% |
|  | S20d_3_Abo | 51.21M | 7.68G | 50.55M | 7.26G | 94.46% | 95.63% | 51.35% |
|  | S60d_1_Abo | 51.68M | 7.75G | 51.03M | 7.34G | 94.72% | 95.66% | 50.70% |
|  | S60d_2_Abo | 49.63M | 7.44G | 48.96M | 7.01G | 94.18% | 95.65% | 50.98% |
|  | S60d_3_Abo | 51.23M | 7.68G | 50.53M | 7.25G | 94.33% | 95.60% | 50.79% |
|  | S15m_1_Abo | 51.21M | 7.68G | 50.56M | 7.22G | 93.97% | 95.66% | 49.54% |
|  | S15m_2_Abo | 51.69M | 7.75G | 51.07M | 7.30G | 94.21% | 95.95% | 48.65% |
|  | S15m_3_Abo | 50.16M | 7.52G | 49.57M | 7.09G | 94.25% | 96.03% | 49.54% |
|  | Adult_1_Abo | 49.18M | 7.38G | 48.61M | 6.98G | 94.64% | 96.00% | 48.46% |
|  | Adult_2_Abo | 51.20M | 7.68G | 50.60M | 7.25G | 94.33% | 95.86% | 49.98% |
|  | Adult_3_Abo | 49.82M | 7.47G | 49.26M | 7.05G | 94.30% | 96.01% | 48.80% |
