# Supplementary material for: Transcriptome profiling in rumen, reticulum, omasum, and abomasum tissues during the developmental transition of pre-ruminant to the ruminant in yaks
Source: Front Vet Sci. 2023 Sep 22;10:1204706. doi: 10.3389/fvets.2023.1204706 (PMC10556492; doi:10.3389/fvets.2023.1204706)
Supplement: Supplementary file 1 [file Data_Sheet_1.zip › Supplemental Materials-0826/Table S1-S22/Table S3-KEGG enrichment analysis of mRNAs in closed groups in rumen.docx]

| **Table S3.** KEGG enrichment analysis of mRNAs in closed groups in rumen (20 d vs. 0 d, 60 d vs. 0 d, 15 m vs. 0 d, and adult vs. 0 d) | |
| --- | --- |
| **Groups** | **Upregulated** |
| 20 d vs. 0 d | Steroid biosynthesis  Steroid hormone biosynthesis  Pentose and glucuronate interconversions  Progesterone-mediated oocyte maturation  Ovarian steroidogenesis  Ascorbate and aldarate metabolism  Hematopoietic cell lineage  Folate biosynthesis  Butanoate metabolism  Mineral absorption  Drug metabolism - other enzymes  Glycosphingolipid biosynthesis - lacto and neolacto series  Arachidonic acid metabolism  Cell cycle  Oocyte meiosis  PPAR signaling pathway  Mucin type O-glycan biosynthesis  Valine, leucine and isoleucine degradation  Ether lipid metabolism  Porphyrin and chlorophyll metabolism |
| 60 d vs. 0 d | Valine, leucine and isoleucine degradation  Metabolism of xenobiotics by cytochrome P450  Steroid hormone biosynthesis  Drug metabolism - other enzymes  Ovarian steroidogenesis  Drug metabolism - cytochrome P450  Propanoate metabolism  Retinol metabolism  Pentose and glucuronate interconversions  Chemical carcinogenesis - DNA adducts  Influenza A  Sulfur metabolism  Folate biosynthesis  Ascorbate and aldarate metabolism  Epstein-Barr virus infection  Butanoate metabolism  Peroxisome  Glutathione metabolism  Arachidonic acid metabolism  Glyoxylate and dicarboxylate metabolism |
| 15 m vs. 0 d | Valine, leucine and isoleucine degradation  Propanoate metabolism  Steroid biosynthesis  Peroxisome  Viral protein interaction with cytokine and cytokine receptor  Cytokine-cytokine receptor interaction  Hematopoietic cell lineage  Pentose and glucuronate interconversions  Drug metabolism - other enzymes  Lysosome  Drug metabolism - cytochrome P450  Glyoxylate and dicarboxylate metabolism  Metabolism of xenobiotics by cytochrome P450  Inflammatory bowel disease  Pyruvate metabolism  Steroid hormone biosynthesis  Ascorbate and aldarate metabolism  Staphylococcus aureus infection  Primary immunodeficiency  Citrate cycle (TCA cycle) |
| Adult vs. 0 d | Valine, leucine and isoleucine degradation  Propanoate metabolism  Metabolism of xenobiotics by cytochrome P450  Drug metabolism - cytochrome P450  Citrate cycle (TCA cycle)  Pyruvate metabolism  Steroid hormone biosynthesis  Chemical carcinogenesis - DNA adducts  Drug metabolism - other enzymes  Glyoxylate and dicarboxylate metabolism  Retinol metabolism  Glutathione metabolism  Peroxisome  Pentose and glucuronate interconversions  Steroid biosynthesis  Hematopoietic cell lineage  Folate biosynthesis  Ovarian steroidogenesis  Ascorbate and aldarate metabolism  Fatty acid degradation |
