# Supplementary figures and images for: Transcriptome profiling in rumen, reticulum, omasum, and abomasum tissues during the developmental transition of pre-ruminant to the ruminant in yaks
Source: Front Vet Sci. 2023 Sep 22;10:1204706. doi: 10.3389/fvets.2023.1204706 (PMC10556492; doi:10.3389/fvets.2023.1204706)

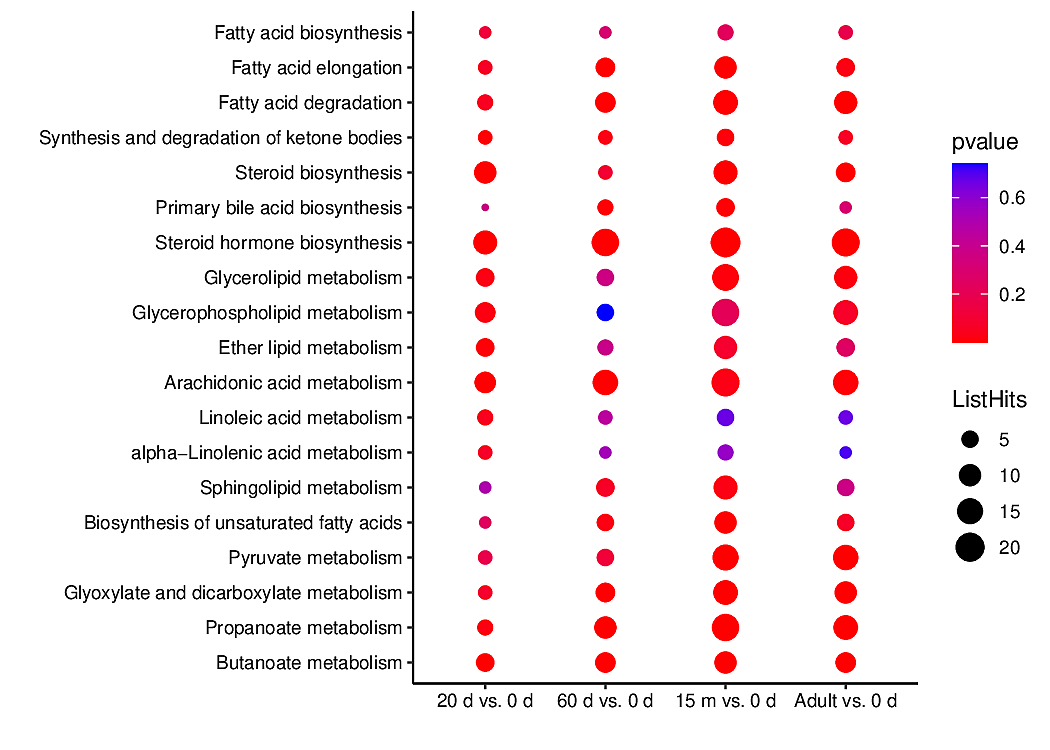

Supplement: Supplementary file 1 [file Data_Sheet_1.zip › Supplemental Materials-0826/Figure S1-S6/Figure S1.png]

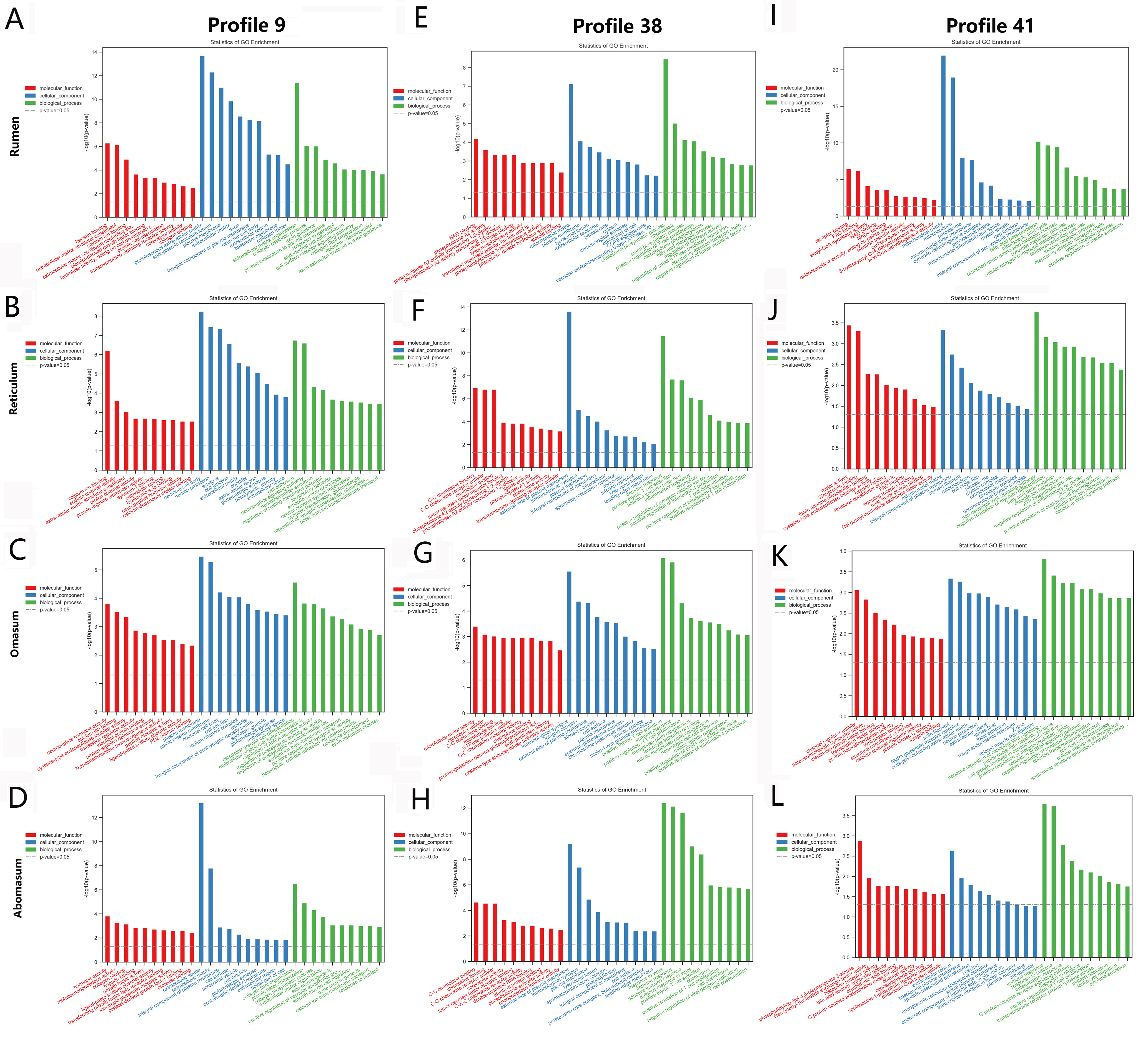

Supplement: Supplementary file 1 [file Data_Sheet_1.zip › Supplemental Materials-0826/Figure S1-S6/Figure S2.jpg]

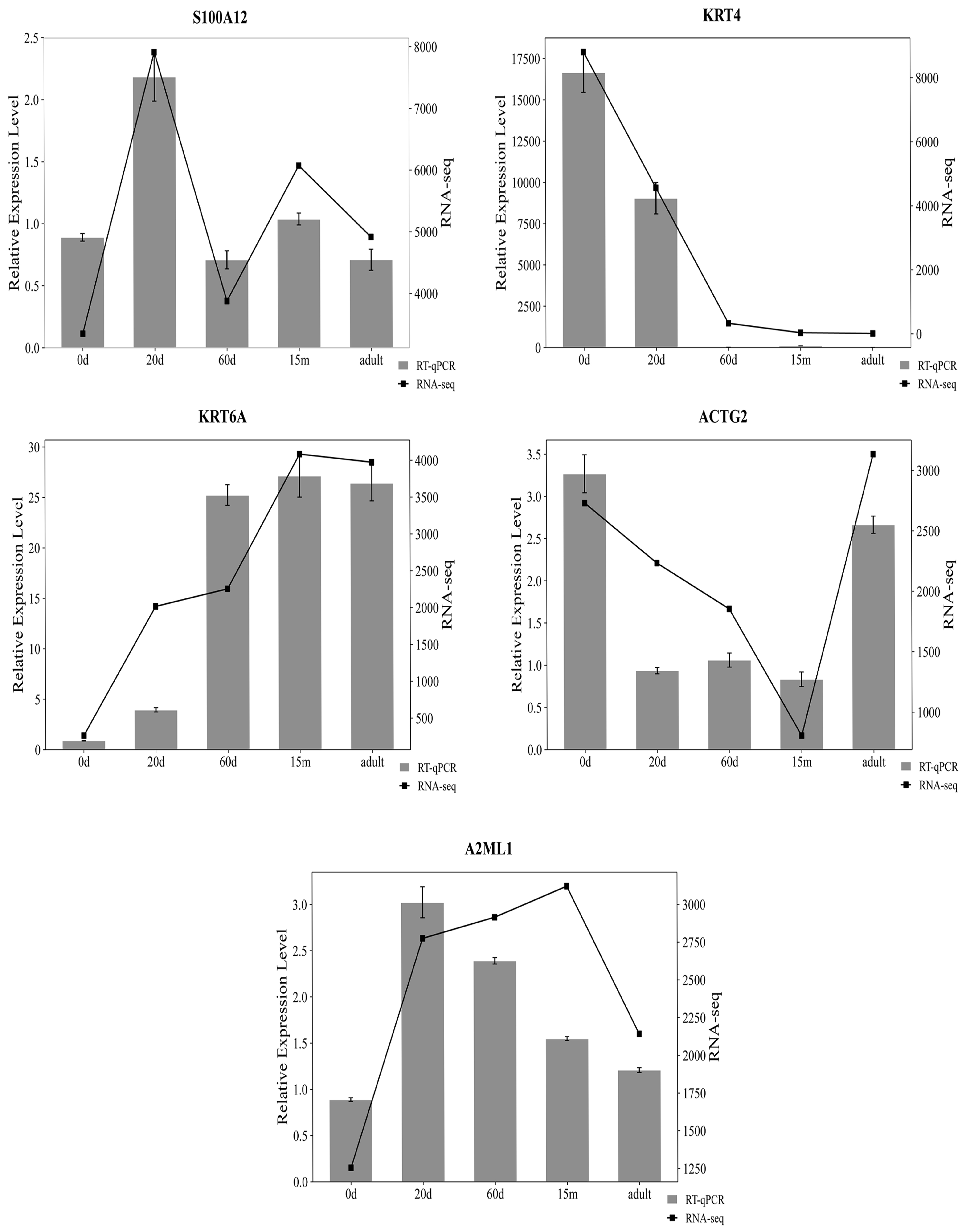

Supplement: Supplementary file 1 [file Data_Sheet_1.zip › Supplemental Materials-0826/Figure S1-S6/Figure S3.jpg]

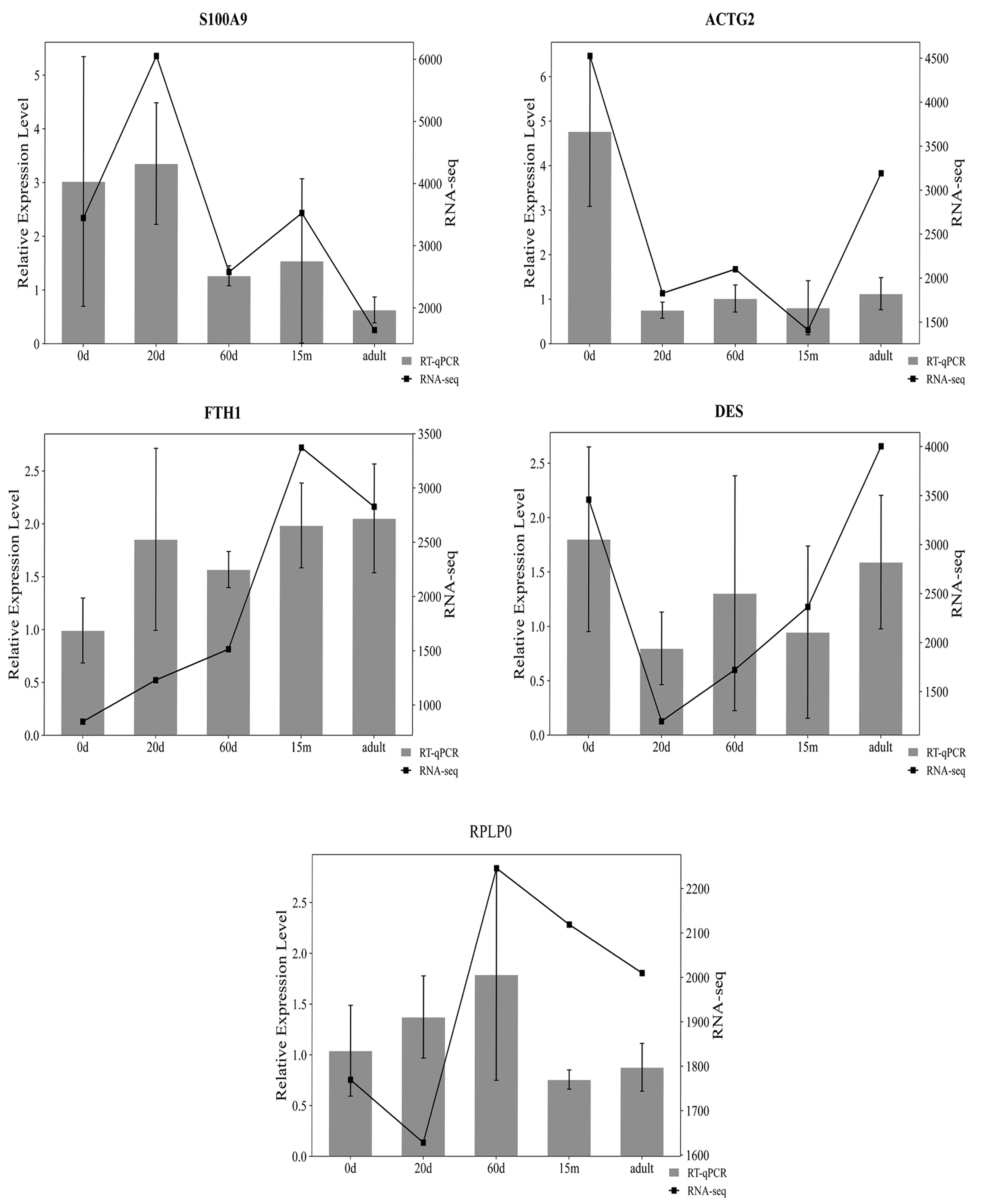

Supplement: Supplementary file 1 [file Data_Sheet_1.zip › Supplemental Materials-0826/Figure S1-S6/Figure S4.jpg]

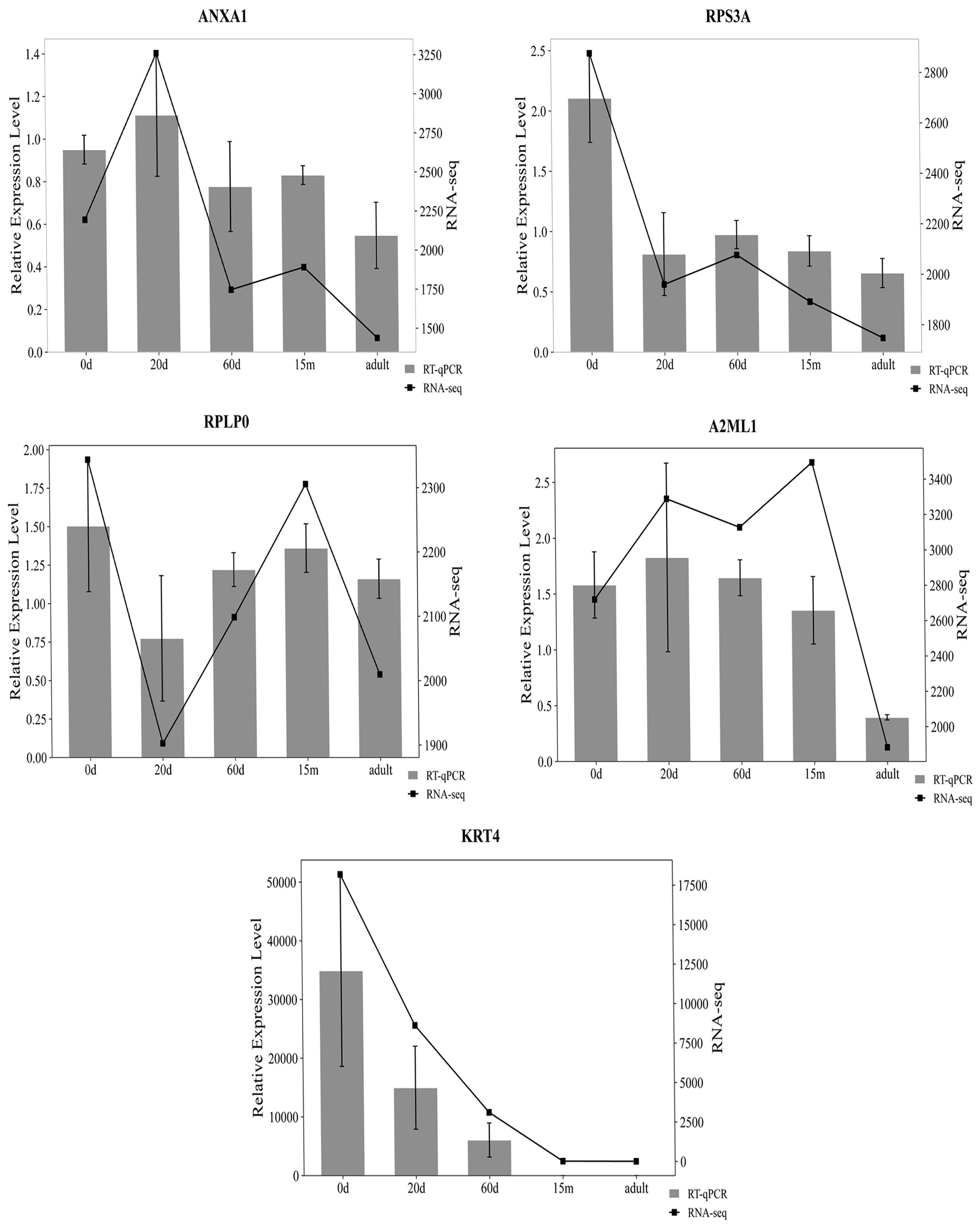

Supplement: Supplementary file 1 [file Data_Sheet_1.zip › Supplemental Materials-0826/Figure S1-S6/Figure S5.jpg]

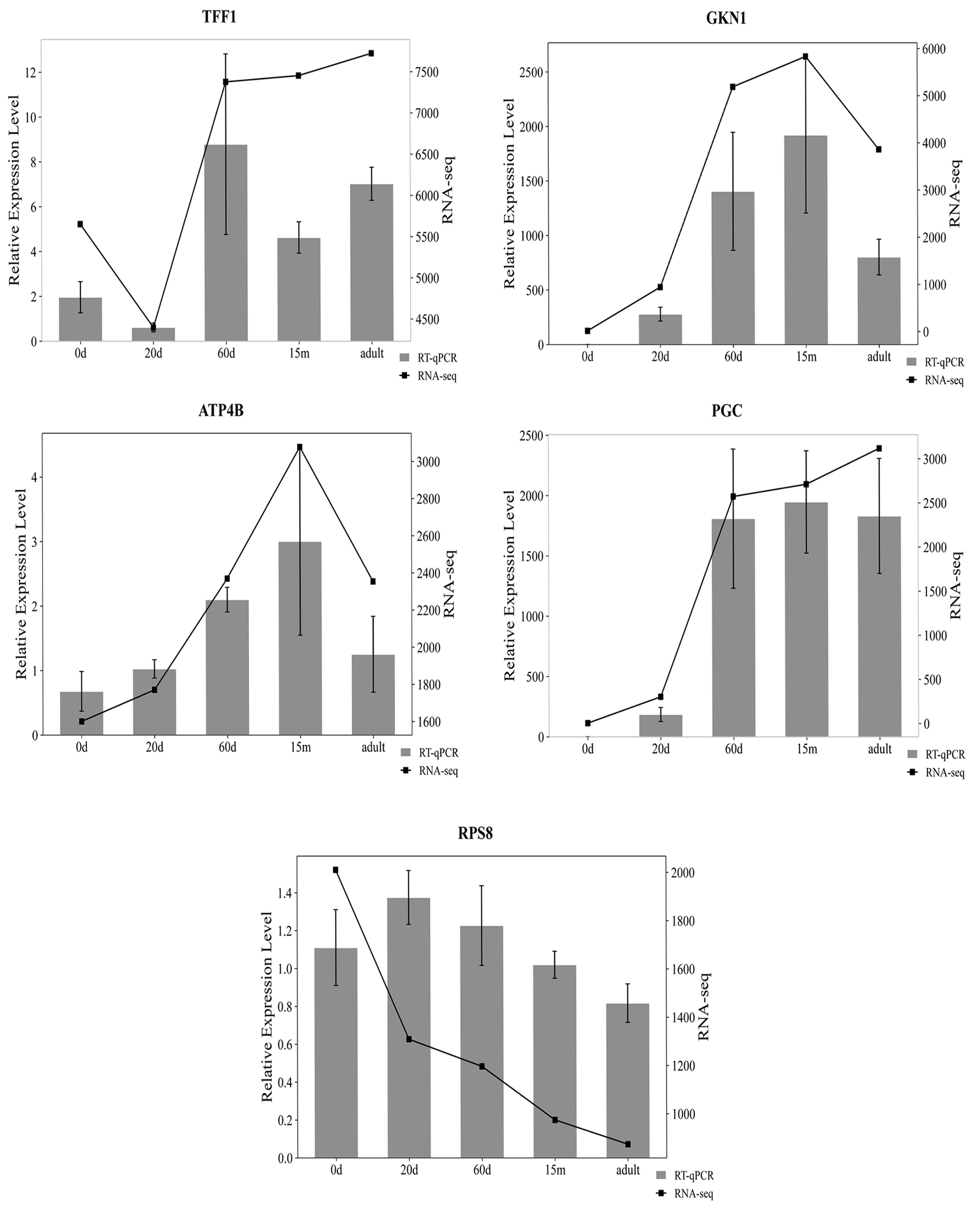

Supplement: Supplementary file 1 [file Data_Sheet_1.zip › Supplemental Materials-0826/Figure S1-S6/Figure S6.jpg]
